# Meningeal lymphatic CGRP signaling governs pain via cerebrospinal fluid efflux and neuroinflammation in migraine models

**DOI:** 10.1172/JCI175616

**Published:** 2024-05-14

**Authors:** Nathan P. Nelson-Maney, László Bálint, Anna L.S. Beeson, D. Stephen Serafin, Bryan M. Kistner, Elizabeth S. Douglas, Aisha H. Siddiqui, Alyssa M. Tauro, Kathleen M. Caron

**Affiliations:** Department of Cell Biology and Physiology, the University of North Carolina at Chapel Hill, Chapel Hill, North Carolina, USA.

**Keywords:** Cell biology, Vascular biology, G protein&ndash;coupled receptors, Lymph, Pain

## Abstract

Recently developed antimigraine therapeutics targeting calcitonin gene–related peptide (CGRP) signaling are effective, though their sites of activity remain elusive. Notably, the lymphatic vasculature is responsive to CGRP signaling, but whether meningeal lymphatic vessels (MLVs) contribute to migraine pathophysiology is unknown. Mice with lymphatic vasculature deficient in the CGRP receptor (*Calcrl^iLEC^* mice) treated with nitroglycerin-mediated (NTG-mediated) chronic migraine exhibit reduced pain and light avoidance compared with NTG-treated littermate controls. Gene expression profiles of lymphatic endothelial cells (LECs) isolated from the meninges of *Rpl22^HA/+^;Lyve1^Cre^* RiboTag mice treated with NTG revealed increased MLV-immune interactions compared with cells from untreated mice. Interestingly, the relative abundance of mucosal vascular addressin cell adhesion molecule 1–interacting (MAdCAM1-interacting) CD4^+^ T cells was increased in the deep cervical lymph nodes of NTG-treated control mice but not in NTG-treated *Calcrl^iLEC^* mice. Treatment of cultured hLECs with CGRP peptide in vitro induced vascular endothelial–cadherin (VE-cadherin) rearrangement and reduced functional permeability. Likewise, intra cisterna magna injection of CGRP caused rearrangement of VE-cadherin, decreased MLV uptake of cerebrospinal fluid (CSF), and impaired CSF drainage in control mice but not in *Calcrl^iLEC^* mice. Collectively, these findings reveal a previously unrecognized role for lymphatics in chronic migraine, whereby CGRP signaling primes MLV-immune interactions and reduces CSF efflux.

## Abstract 2

CGRP primes meningeal lymphatic vessels to interact with immune cells, promotes VE-cadherin continuous junction formation, and reduces meningeal lymphatic vessel permeability, resulting in migraine pain.

## Graphical abstract

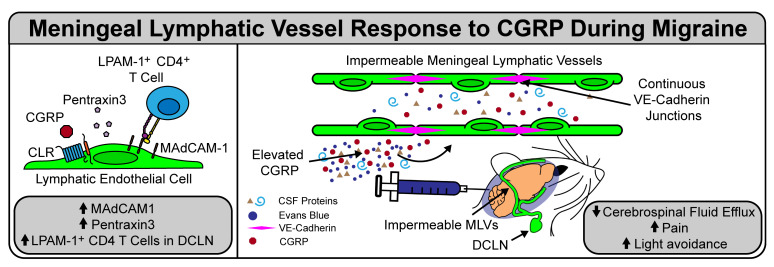

## Introduction

The meninges have traditionally been considered a protective and physical barrier to the brain, shielding the central nervous system from external trauma or systemic insults such as infection and inflammation. More recently, a complex network of meningeal lymphatic vessels (MLVs) within the dura mater layer of the meninges have been studied intensely, revealing unexpected and critical functions for the meninges in normal cerebrospinal fluid (CSF) turnover (1, 2) and in pathological conditions such as Alzheimer’s disease, Parkinson’s disease, aging, and neuroinflammation (3–6). Mice also have MLVs that course along the superior sagittal sinus and transverse sinus on the dorsal aspect of the cranium and along the petrosquamosal and sigmoid sinuses, including localizations near the retroglenoid vein and pterygopalatine artery on the basal aspect of the cranium (1, 2, 7). Drainage of CSF to the deep cervical lymph nodes (DCLNs) can be visualized by intra cisterna magna (ICM) injection of tracers into the CSF (3, 7, 8), validating the physiological function of MLVs in CSF drainage. The lymphatic system also functions as a conduit for immune cells and antigens from peripheral tissues to the tissue draining lymph nodes using well-established chemotactic gradients such as CCL21 and CCL27 to attract CCR7 and/or CCR10-expressing immune cells (9). This CCR7-CCL21 immune cell trafficking was observed to occur in a mouse model of multiple sclerosis, validating that this well-established paracrine signaling is present in the MLVs as well. Therefore, it is surprising that only a few studies have begun to explore the involvement of MLVs in the highly prevalent condition of chronic migraine (10).

Chronic migraine is a pain syndrome that has neuronal, vascular, and immunologic components (11) and is classically mediated by elevated levels of the neuropeptide calcitonin gene-related peptide (CGRP) in both plasma (12–14) and CSF (15–17). CGRP is a potent vasodilatory neuropeptide that is released from trigeminal C–fibers during migraine (18, 19) and is hypothesized to act upon dural and cerebral vessels, including the superior sagittal sinus (an anatomical location of the dorsal MLVs) to cause vasodilation and consequent stimulation of immune cells (20). This meningeal neurogenic inflammation affects cells from both innate and adaptive immune responses, provoking inflammatory responses including Il-1β, Il-6, TNF-α, leukotrienes, prostaglandins, soluble VCAM-1, and soluble ICAM-1, as well as transient blood brain barrier breakdown allowing for immune cell transmigration into the CNS (21–24). Meningeal neurogenic inflammation is also hypothesized to support a feed-forward pain pathway, contributing to the overall pathophysiology of migraine, with the trigeminal ganglion serving as a critical component of the ascending CNS pain pathways to the thalamus and hypothalamus and the dorsal horn of the spinal cord, playing a central role in the onset of ensuing headache pain (25). Nonsteroidal antiinflammatory drugs (NSAIDS) are effective as a migraine abortive medication and are often first choice medications to treat migraine acutely. However, NSAIDS are ineffective in a large proportion of patients with migraine, are not recommended for chronic use, and migraine therapeutics generally do not target inflammation (11). Consequently, several new medications recently approved by the FDA target the CGRP signaling axis and are effective at reducing both the duration and intensity of migraine pain: monoclonal antibodies to the CGRP receptor (erenumab), monoclonal antibodies to plasma CGRP (galcanezumab, fremanezumab, and eptinezumab), and small molecule antagonists for the CGRP receptor (26).

Though these novel antibody medications targeting CGRP and the CGRP receptor have shown promise in migraine treatment, their precise sites of action—which, according to the pharmacodynamics of monoclonal antibodies, must reside outside the blood brain barrier—remain poorly understood. As a potent vasodilator and vascular permeability agent, CGRP targets the smooth muscle and endothelial cells of large cerebral blood vessels of the meninges, suggesting that some of the therapeutic effectiveness of anti-CGRP medications is imparted through modulation of the vasculature (21). The CGRP receptor is a heterodimer formed from Calcitonin-receptor like-receptor (gene: *Calcrl*, protein: CLR) and receptor activity modifying protein 1 (gene: *Ramp1*, protein: Ramp1), and, interestingly, is expressed at higher levels in lymphatic endothelial cells (LECs) compared with blood endothelial cells (27, 28). Consistently, mice and humans with homozygous loss-of-function mutations in the gene that encodes CLR die in utero from nonimmune hydrops fetalis and arrested developmental lymphangiogenesis (29, 30). Lymphatic-specific deletion of *Calcrl* in mice recapitulates the edematous hydrops fetalis phenotype of global *Calcrl* deletion, demonstrating the requisite role for CLR signaling in lymphatic development. In adult animals, tamoxifen-mediated deletion of *Calcrl* in lymphatics causes impaired intestinal lymphatic vessel (LV) lipid uptake and intestinal lymphangiectasia (31, 32), illustrating the necessity of sustained CLR signaling in maintaining lymphatic structure and function during adulthood. Moreover, global loss of the *Ramp1* gene, which encodes a requisite chaperone protein required for CGRP signaling (33–36), also causes impaired lymphatic growth and function (37). Collectively, these data support a principal role for CGRP receptor signaling in lymphatics during development and adulthood.

At the cellular level, activation of the CLR G protein–coupled receptor pathway results in increased MAPK/ERK and pAKT downstream signaling in LECs, culminating in cellular proliferation (38). CLR signaling can also support the transactivation of other important, growth-promoting lymphatic signaling pathways, such as vascular endothelial growth factor-C (VEGFC)/VEGFR3 signaling, both in cultured cells and in vivo (39). Finally, activation of CLR stimulates the rapid and robust reorganization of LEC junctional and gap proteins, like vascular endothelial–cadherin (VE-cadherin), zonula occludens-1, and connexins, to tighten intercellular barriers and reduce lymphatic permeability (30, 40, 41). Most of these cellular effects of CLR signaling have been attributed to the activation of the CLR-RAMP2 heterodimer by adrenomedullin (AM) ligand, leaving open the possibility that migraine-induced CGRP ligand may elicit similar effects through CLR-RAMP1 heterodimers in LECs of MLVs.

There are several animal models of chronic migraine that faithfully recapitulate many aspects of human chronic migraine pathophysiology, including elevations in CGRP (42–44). Some migraine models, including direct stimulus of cortical spreading depression require invasive procedures including burrhole craniotomy or skull thinning, potentially damaging the delicate underlying meninges or MLVs or provoking trauma-induced inflammatory responses (12, 42, 43). Peripheral or central administration of CGRP remains a widely used and translationally relevant model of migraine in multiple species with resultant systemic vasodilation, peripheral allodynia, hyperalgesia, periorbital hypersensitivity, and immobile behaviors (42, 43). Finally, i.p. injection of nitroglycerin (NTG), a potent NO donor, sensitizes the trigeminovascular system to cause the release of endogenous CGRP from trigeminal ganglion neurons, evoking migraine-like pain and associated behaviors in rodents, including aversion to light and hyperalgesia in response to light touch (44–46). Humans taking NTG for clinical use have reported migraine-like episodes similar to spontaneous migraines, mimicking neural activity in a spontaneous migraine, though it is still unclear how much this model depends on CGRP in humans (46). Therefore, in this study, we chose to use cell culture models, nonsurgical NTG injections, and intrathecal CGRP injections in genetic mouse models to evaluate whether and to what extent chronic migraine-induced elevations in CGRP influence MLVs and subsequent migraine pathophysiology.

## Results

### Lymphatic-specific deletion of the Calcrl gene, encoding the CGRP receptor, alleviates facial expression of chronic migraine pain.

To test whether MLVs contribute to the pathophysiology of chronic migraine pain, *Calcrl^fl/fl^*;*Vegfr3^CreERT2/+^* (*Calcrl^iLEC^*) mice with lymphatic-specific, temporal genetic deletion of the CGRP receptor, were evaluated for facial pain following NTG induction of chronic migraine. *Vegfr3^CreERT2^* mice have been previously described and are commonly used as a tool to modify genes in lymphatics, and, depending on timing, copy number and dosage, in some capillary blood endothelial cells of some organs (47). To assess the extent of the tamoxifen-mediated deletion, expression of *Calcrl* 2 weeks after tamoxifen injection was significantly reduced by 60% in meningeal LECs isolated from *Calcrl^iLEC^* mice compared with those of *Calcrl^fl/fl^* mice (0.413 ± 0.19 SD, *P* = 0.0063, unpaired student’s *t* test). *Calcrl* mRNA levels were unchanged in Lyve1^–^, CD68^–^ cells isolated from cardiac tissue of *Calcrl^iLEC^* mice compared with those of *Calcrl^fl/fl^* mice (1.8 ± 1.6 SD, not significant by student’s *t* test).

**Figure 1:**
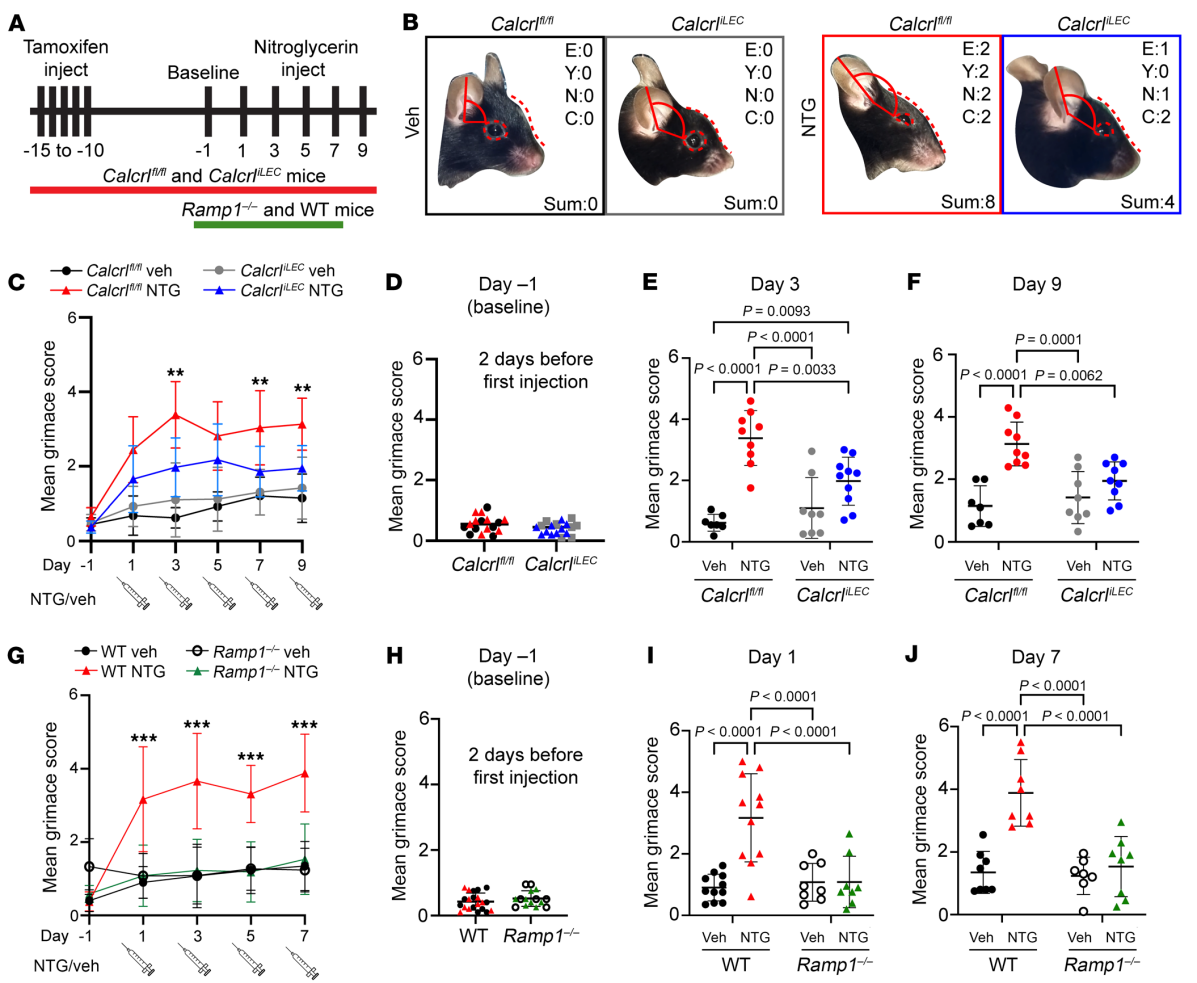
*Calcrl^iLEC^* mice treated with NTG exhibit partially ameliorated chronic migraine pain. (**A**) Experimental protocol representation. (**B**) Images of mouse facial expression of pain on Day 6, minute 42 following injection. Unit scores are depicted. E, ears; Y, eyes; N, nose; C, cheek. Sum of scores tallied bottom right. (**C**) Facial expression of pain measured 30 minutes after NTG injection and recorded for 20 minutes. Facial expression of pain is scored once per minute for the 20-minute recording duration and averaged. Mice were allowed to acclimate to the chamber 4 days before first injection (day 1) and 2 days before baseline measurement (day –1). **P < 0.01 between *Calcrl^fl/fl^* and *Calcrl^iLEC^* treated with NTG. Mean grimace score on (**D**) day –1 (preinjection baseline) grouped by genotype. Colors indicate injection given later in experimental protocol, matching panel **B**. Mean Grimace Score on (**E**) day 3, and (**F**) day 9 of chronic migraine model. For **C**–**F**, *n* = 7–10 animals per group representing 4 independent cohorts. Significance calculated using 2-way ANOVA with Tukey’s multiple comparisons test. Graphs show mean ± SD. (**G**) Facial expression of pain for *Ramp1^–/–^* animals and controls. ***P < 0.001 between *Ramp1^–/–^* and WT treated with NTG. Mean grimace score on (**H**) day –1 (preinjection baseline) grouped by genotype. Colors indicate injection given later in experimental protocol, matching key in panel **G**. Not all animals were recorded for baseline. Mean grimace score on (**I**) day 1, and (**J**) day 7 of chronic migraine model. For **G**–**J**, *n* = 8–11 animals per group representing 3 independent cohorts. Significance calculated using 2-way ANOVA with Tukey’s multiple comparisons test. Graphs show mean ± SD.

In this study, chronic migraine was induced by i.p. injection of NTG every other day for 8 days (5 total injections) and compared with animals with 0.9% normal saline injections (Figure 1, A and B). This model has been demonstrated to be primarily mediated by CGRP (44–46), and we confirmed an increase in CGRP peptide levels adjacent to meningeal lymphatics following NTG injection (Supplemental Figure 2). Pain behavior was quantified 30 minutes after each injection using the well-established murine grimace scale (48), which incorporates changes in ear, eye, nose, and cheek positions as a surrogate measure of pain (Figure 1B). Mean grimace scores (MGSs) were calculated from video recordings once every minute for 20 minutes and averaged to generate a MGS (Figure 1, A and B). As we assigned 0–2 total points to 4 clinical categories, mice could score between 0 points (no pain at all) and 8 points (most possible pain detectable, such as surgical pain without analgesia).

There were no significant differences in facial characteristics of pain between *Calcrl^fl/fl^* and *Calcrl^iLEC^* mice at baseline 1 day before NTG injection (Figure 1, C and D). As expected, NTG-treated *Calcrl^fl/fl^* mice exhibited significantly higher MGSs compared with vehicle-treated littermates (Figure 1, B and C), with peak values at Day 3 (Figure 1, C and E) that remained chronically elevated through Day 9 (final day of testing) (Figure 1, C and F). Interestingly, *Calcrl^iLEC^* mice with lymphatic loss of the CGRP receptor exhibited significantly attenuated MGS after treatment with NTG compared with *Calcrl^fl/fl^* littermates treated with NTG (Figure 1C). Throughout the testing period, *Calcrl^iLEC^* mice treated with NTG exhibited average MGSs ranging from 1.8 to 2.2, which did not differ significantly at any point from vehicle-injected *Calcrl^iLEC^* mice (Figure 1, D–F). Success of the lymphatic-specific deletion of *Calcrl* was confirmed via immunoprecipitation and qPCR, which indicated approximately 70% deletion of *Calcrl* in MLVs (Supplemental Figure 1A; supplemental material available online with this article; https://doi.org/10.1172/JCI175616DS1). Changes to the lymphatic vascular network in the meninges 3 weeks after tamoxifen-induced deletion was assessed using immunofluorescence microscopy. Using AngioTool, it was determined that there was no difference in vessel area, vessel length, vessel branchpoints, or vessel endpoints (Supplemental Figure 1, B–E).

To confirm whether the observations made in *Calcrl^iLEC^* mice were mediated by CGRP, chronic migraine-like pain was also assessed in *Ramp1^–/–^* mice using the NTG-mediated model of chronic migraine following the same protocol as above. These animals were only assessed to day 7, due to experimental endpoint constraints. There were no significant differences in facial characteristics of pain between WT control and *Ramp1^–/–^* mice at baseline 1 day before NTG injection (Figure 1, G and H). Consistent with our findings in NTG-treated *Calcrl^fl/fl^* mice, NTG treated WT controls exhibited significantly higher MGSs compared with vehicle-treated littermates (Figure 1, G, I, and J), with peak values at Day 7 that remained chronically elevated through the extent of testing (Figure 1, G and J). As expected, mice that are systemically deficient in *Ramp1* exhibited completely normal MGS after treatment with NTG compared with WT controls treated with NTG (Figure 1G), as these animals have no tissues that express *Ramp1*, a required component for the highest affinity CGRP receptor. Throughout the testing period, *Ramp1^–/–^* mice treated with NTG exhibited average MGS ranging from 1.4 to 1.7, which did not differ significantly at any point from vehicle-injected mice (Figure 1, G–J).

Together, these data indicate that expression of the CGRP receptor *Calcrl* in LVs contributes to the manifestation of chronic migraine pain induced by NTG injection in mice. These data also indicate that the NTG model of chronic migraine is primarily mediated through CGRP signaling through it’s primary receptor complex, CLR in complex with Ramp1.

### Light avoidance and anxiety-like behavior is abrogated in Calcrl^iLEC^ mice treated with chronic migraine–inducing NTG.

**Figure 2:**
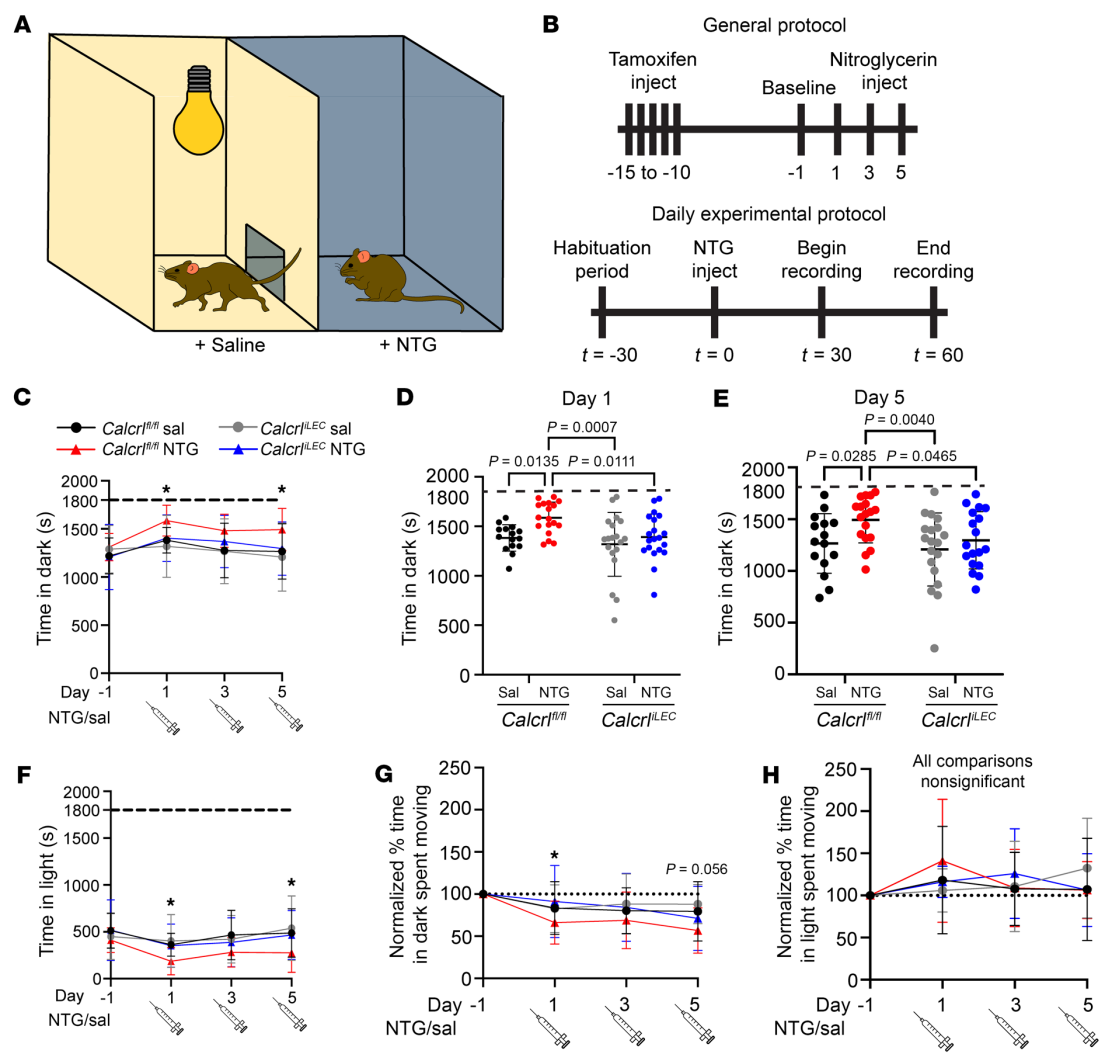
*Calcrl^iLEC^* mice treated with NTG exhibit ameliorated light avoidance and anxiety behavior. (**A**) Schematic of light aversion and movement assay. (**B**) Schematic of general (top) and daily (bottom) experimental protocol. Mice were allowed to acclimate to the chamber 4 days before the first injection and 2 days before the baseline measurement (baseline measurement recorded on day –1) Light avoidance and movement behavior are measured 30 minutes after NTG injection and were recorded for 30 minutes. (**C**) Time spent in dark chamber reported for all test days and on day 1 (**D**) and day 5 (**E**). Dashed line shows the total test time (1,800 seconds or 30 minutes). (**F**) Time spent in light chamber reported for all test days. (**G**) Normalized percent time spent moving in the dark chamber and (**H**) normalized percent time spent in moving in the light chamber for all experimental days. Percent of time spent moving in the respective chamber were moving normalized to baseline movement in the same chamber data. Data are normalized to the baseline measurement on day –1 by individual calculated as percent time moving on test day divided by baseline percent time moving (day –1) multiplied by 100. *P < 0.05, **P < 0.01 reported between *Calcrl^fl/fl^* and *Calcrl^iLEC^* treated with NTG. Dashed line, baseline. **C**–**H**, *n* = 14–20 animals per group. Data represents 12 independent cohorts. Significance for all data calculated using 2-way ANOVA with Tukey’s multiple comparisons test. *P* values indicated if < 0.1. Graphs = mean ± SD.

Another clinical hallmark of migraine is aversion to sensory stimuli such as bright lights or strong odors. To assess the involvement of lymphatic CGRP signaling on migraine behavior in a second assay complementary to pain, we assessed light aversion and movement behavior. Mice undergoing the chronic NTG–induced chronic migraine protocol described above were placed in a VersaMax light aversion/activity recording apparatus consisting of a bright and dark chamber, with free access between chambers (Figure 2A). Daytime video recordings during the natural nocturnal period were evaluated to quantify the percent of time that mice were moving in the dark chamber, as indicated by breaks within a grid of laser beams. Light avoidance behavior was assessed 2 days before the first injection on day –1, and then following NTG injection on days 1, 3, and 5 of the experimental protocol (Figure 2B). On each testing day, animals were allowed to acclimate to the testing room for 30 minutes prior to injection. Animals were then injected with 10 mg/kg NTG and allowed to rest for an additional 30 minutes. Light avoidance behavior was recorded for 30 minutes (Figure 2B). Due to limitations of the darkened chamber’s construction, we were unable to assess rearing behaviors. Typical of mouse behavior during the nocturnal period, we found that all mice spent a majority of the test time in the dark chamber (Figure 2C).

There were no significant differences in light avoidance or movement between *Calcrl^fl/fl^* and *Calcrl^iLEC^* mice at baseline 1 day before NTG injection (Figure 2C). Significant increases in time spent in the dark were observed on days 3 and 5 of testing between saline *Calcrl^fl/fl^* and NTG-treated *Calcrl^fl/fl^* mice. Correlating with our facial expression of pain observations, NTG-treated *Calcrl^iLEC^* mice demonstrated reduced light avoidance behaviors when compared with NTG-treated *Calcrl^fl/fl^* mice on days 3 and 5 (Figure 2, C–F).

Activity level while in the dark is an additional appropriate measure that integrates light aversion, movement, and anxiety behavioral measurements for assessing pain intensity (49). Chronic migraine induced by NTG injection caused a significant attenuation of relative movement in the dark chamber in NTG-treated *Calcrl^fl/fl^* mice compared with vehicle-treated *Calcrl^fl/fl^* mice on days 3 and 5. Importantly, throughout the testing period no significant difference in light avoidance or relative time moving was observed between *Calcrl^iLEC^* mice injected with NTG and *Calcrl^fl/fl^* or *Calcrl^iLEC^* mice on days 1, 3, and 5 (Figure 2, G and H), indicating that loss of CGRP signaling in lymphatics attenuates chronic migraine–associated behaviors to a level observed in saline-injected animals. *Calcrl^iLEC^* mice exhibited significantly different movement in the dark behaviors compared with NTG-treated *Calcrl^fl/fl^* mice on day 1 and on day 5, though the *P* value was calculated to be 0.056 (Figure 2G) indicating that *Calcrl^iLEC^* mice do not experience chronic migraine–associated light aversion or anxiety. The converse phenotype, relative amount of time spent moving in the light, was not found to be significantly different between any of the genotypes or treatments (Figure 2H). These findings are consistent with the results of similar studies of WT mice (50, 51).

Thus, using 2 independent behavioral metrics of chronic migraine pain, facial grimace and light avoidance/movement, these data support the conclusion that CGRP signaling in lymphatics contributes significantly to the pathophysiological presentation of chronic migraine pain.

### MLVs exhibit transcriptional changes in response to NTG-mediated chronic migraine.

**Figure 3:**
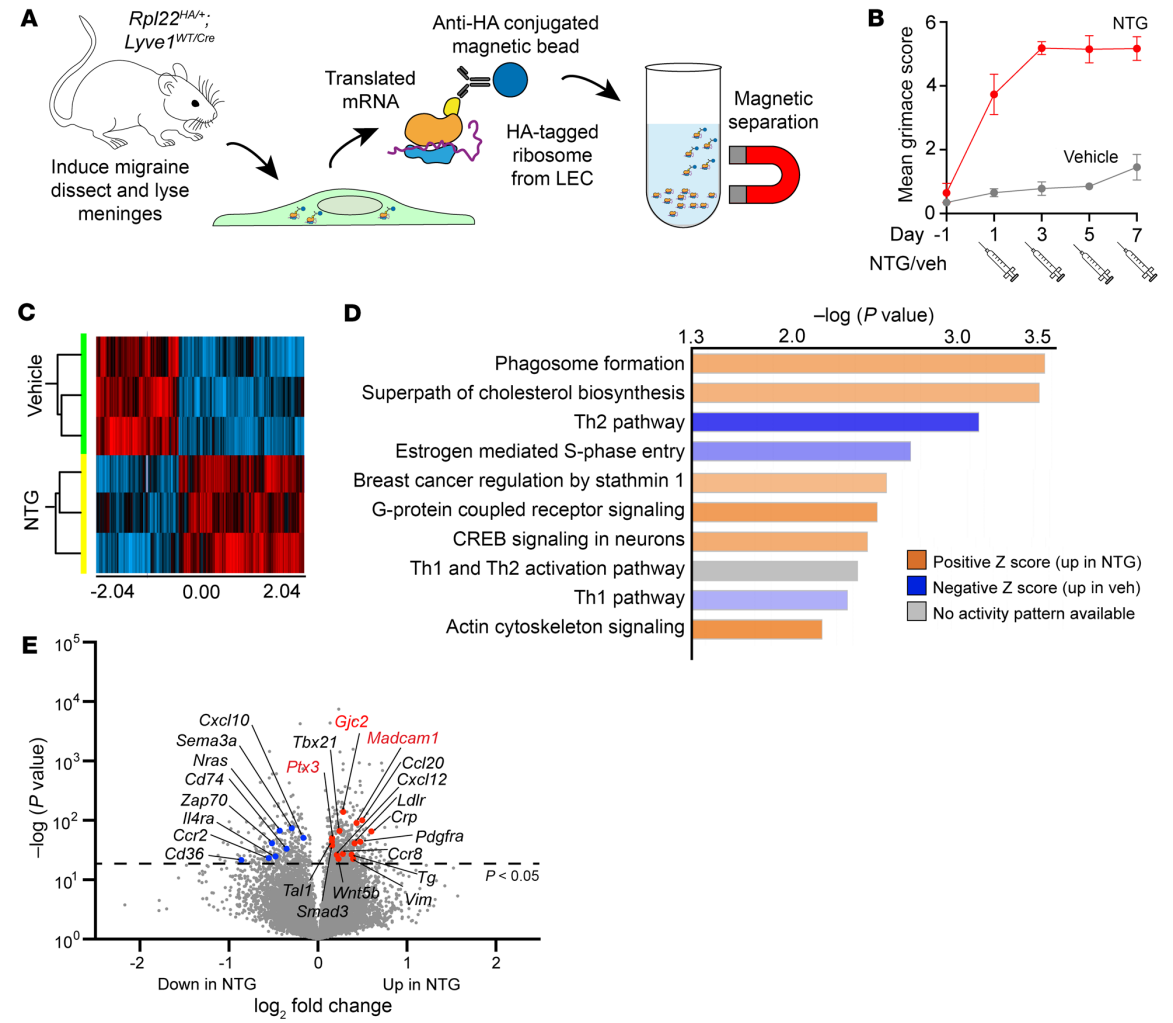
Mice treated with NTG model of chronic migraine exhibit unique MLV translational profiles. (**A**) RiboTag Lyve1-Cre schematic depicting experimental protocol. (**B**) Mean Grimace Score for RiboTag mice, confirming initiation of chronic migraine model. *n* = 3 animals per group. Data not recorded from final injection day (Day 8). Graph shows mean ± SD. (**C**) Heatmap of differentially translated genes. Red, up with NTG; Blue, down with NTG. *n* = 3 animals per group from 1 cohort. (**D**) IPA analysis of differentially expressed genes from microarray. Top 10 pathways with more than 4 identified transcripts per pathway displayed. (**E**) Volcano plot of significant differentially genes in MLVs. Labeled genes were selected because PubMed searches resulted in over 7 papers with the topic trend of lymphatic biology. Red text, upregulated and investigated. Significance determined by 1-way ANOVA. Significant *P* values < 0.05 (above dashed line).

To query and to characterize the genome-wide transcriptional and translational response of meningeal lymphatic vasculature during NTG-mediated chronic migraine we utilized the RiboTag genetic model (Figure 3A) (52). We generated and used *Rpl22^HA/+^;Lyve1^Cre^* mice, which enables the HA-tagging and immunoprecipitation of actively translating mRNAs from Lyve1-Cre^+^ LECs of dissected meninges (Figure 3A). The *Rpl22^HA/+^;Lyve1^Cre^* mice were treated with either vehicle or NTG to induce a state of CGRP elevation and chronic migraine. Prior to meninges dissection, we confirmed that mice receiving NTG injections exhibited significant increases in pain behavior compared with vehicle controls, as quantified by their MGS (Figure 3B).

Clariom S Pico microarray analysis of *Lyve1*-*Cre*^+^ ribosome-associated mRNAs revealed 2 distinct groups of 700 differentially expressed genes between NTG and vehicle-treated mice, calculated using 1-way ANOVA with a FDR of 0.05 (Figure 3C and Supplemental Table 1). Ingenuity Pathway Analysis (IPA, Qiagen) of differentially expressed genes identified NTG-induced increases in the expression of genes associated with phagocytosis, cholesterol biosynthesis, G protein-coupled receptor (GPCR) signaling, cAMP response element-binding protein signaling, and actin cytoskeletal signaling along with NTG-induced decreases in several Th1 and Th2 activation pathways (Figure 3D).

Consistent with the effectiveness of RiboTag to isolate actively translated lymphatic mRNAs from meninges, a high number of genes with previously described roles in lymphatics were identified through bibliometric analysis (Figure 3E, labeled genes), including *Gjc2* and *Cxcl12*, which have been linked to *Calcrl* signaling in lymphatics (40, 53, 54). Interestingly, the data revealed enrichment of 2 established serum biomarkers of chronic migraine, *Crp* (55) and *Ptx3* (56–58), again suggesting the involvement of meningeal lymphatics in chronic migraine pathophysiology. In addition, many chemokine/cytokine and immune signaling genes were identified, including *Ptx3*, *Madcam1*, *Cxcl10*, *Cd74*, *Il4ra*, *Ccr2*, *Cd36*, *Ccl20*, *Cxcl12*, and *Ccr8*. This gene signature is indicative of an immune response within the meninges following NTG treatment and broadly supporting neuro, vascular, and immune crosstalk during chronic migraine responses (11, 23, 59). Because MLVs play significant roles in both immune cell trafficking and the homeostatic efflux of CSF from the CNS, we elected to validate several candidate genes that might play functional roles in either multitissue crosstalk or fluid homeostasis of migraine pathophysiology.

### CGRP treatment of cultured hLECs is sufficient to induce transcriptional changes in differentially translated genes observed in MLVs during NTG-induced chronic migraine.

Since the RiboTag gene expression analysis reflects translational changes in response to systemically administered NTG, we elected to validate that CGRP peptide itself was sufficient to induce changes in gene expression of 3 identified genes using cultured human dermal LECs treated with 100 nM CGRP: Connexin-47 (gene: *Gjc2*, protein: Connexin-47), Pentraxin3 (gene: *Ptx3*, protein: PTX3), and Mucosal vascular addressin cell adhesion molecule 1 (gene: *Madcam1*, protein: MADCAM1) (Figure 3E, red text).

**Figure 4:**
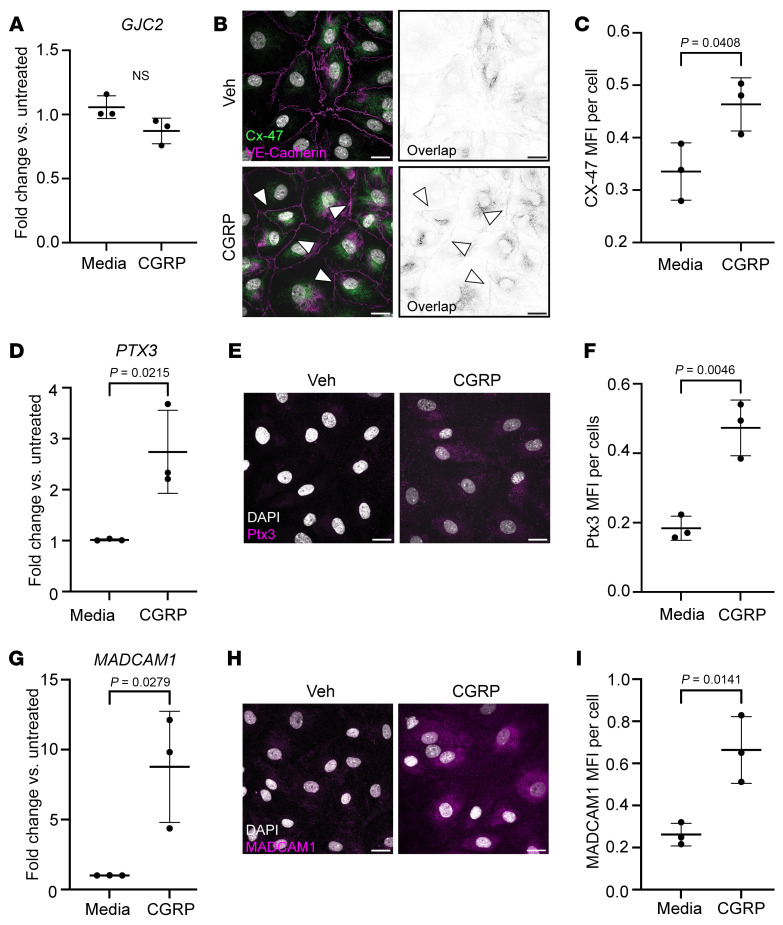
CGRP induces protein level changes in LECs. (**A**) qPCR analysis of *GJC2* in LECs. (**B**) Confocal microscopy of connexin-47 (green) and VE-cadherin (magenta) in vehicle (Veh) and CGRP-treated LECs in vitro. Right, Image of overlapping Connexin-47 and VE-cadherin signal. Black represents Connexin-47 and VE-cadherin colocalization. Pixels have signal only if there is signal for both VE-cadherin and Cx-47. Arrows, connexin-47 at continuous VE-cadherin borders. (**C**) Quantification of MFI of connexin-47. (**D**) qPCR analysis of *PTX3* in CGRP-treated LECs. (**E**) Immunofluorescence of Pentraxin3 in LECs. (**F**) Quantification of MFI of Pentraxin3 in LECs. (**G**) qPCR analysis of *MADCAM1*. (**H**) Immunofluorescence of MADCAM1 in CGRP-treated LECs. (**I**) Quantification of MFI of MADCAM1 in LECs. For all qPCR analysis (**A**, **D**, and **G**), *n* = 3 biological replicates and with 3 technical replicates. For all immunofluorescence experiments (**C**, **F**, and **I**), *n* = 3 biological replicates with 3 randomly selected fields of view averaged for each biological replicate. Significance for all data presented calculated using 2-tailed, unpaired student’s *t* test. Scale bar: 20 μm. Graphs show mean ± SD.

Mutations in the gene encoding for the gap junction protein Cx47 (*GJC2*) have been described as causal variants in primary lymphedema (60, 61); however, the expression of *GJC2* mRNA was not significantly changed in cultured hLECs upon treatment with CGRP for 8 hours (Figure 4A). Cx47 protein levels were significantly, though modestly, upregulated by approximately 1.5 fold 8 hours after CGRP treatment, as quantified by immunofluorescence (Figure 4, B and C). Interestingly, we noted that Cx47 appeared to colocalize along continuous VE-cadherin adherens junctions between LECs — a pattern that was more apparent in the CGRP-treated condition (Figure 4B, white arrowheads).

Pentraxin3 (PTX3) is a secreted, acute phase reactant expressed by many cell types, including within blunt end terminals of lymphatic capillaries (62) and in paracortical and cortical sinus LECs of lymph nodes (63). Moreover, PTX3 is associated with endothelial dysfunction and vascular inflammation and serves as a biomarker of endothelial damage during chronic migraine (57). *PTX3* mRNA expression in human LECs was significantly elevated, 2.5-fold, following 8 hour CGRP treatment in cultured hLECs (Figure 4D). Additionally, PTX3 protein was significantly upregulated, approximately 2-fold, 8 hours following CGRP treatment, as quantified by immunofluorescence (Figure 4, E and F).

MADCAM1 is an endothelial cell adhesion molecule that interacts with α4/β7 integrins expressed on immune cells, facilitating immune cell adhesion to endothelial cells (64). *MADCAM1* mRNA was robustly enriched 9-fold in hLECs following 8 hour CGRP treatment (Figure 4G). MADCAM1 protein was also significantly upregulated, 3-fold, in hLECs after 8 hours of CGRP treatment (Figure 4, H and I). Collectively, these data demonstrate that meningeal LECs in mice with NTG-induced chronic migraine as well as cultured hLECs cells exposed to CGRP peptide exhibit changes in gene and protein expression that are suggestive of lymphatic vascular-immune interactions during chronic migraine pathophysiology.

### NTG-induced CGRP signaling primes MLV capillary endpoints and is required for egress of LPAM1^+^CD4^+^ T cells to draining cervical lymph nodes.

**Figure 5:**
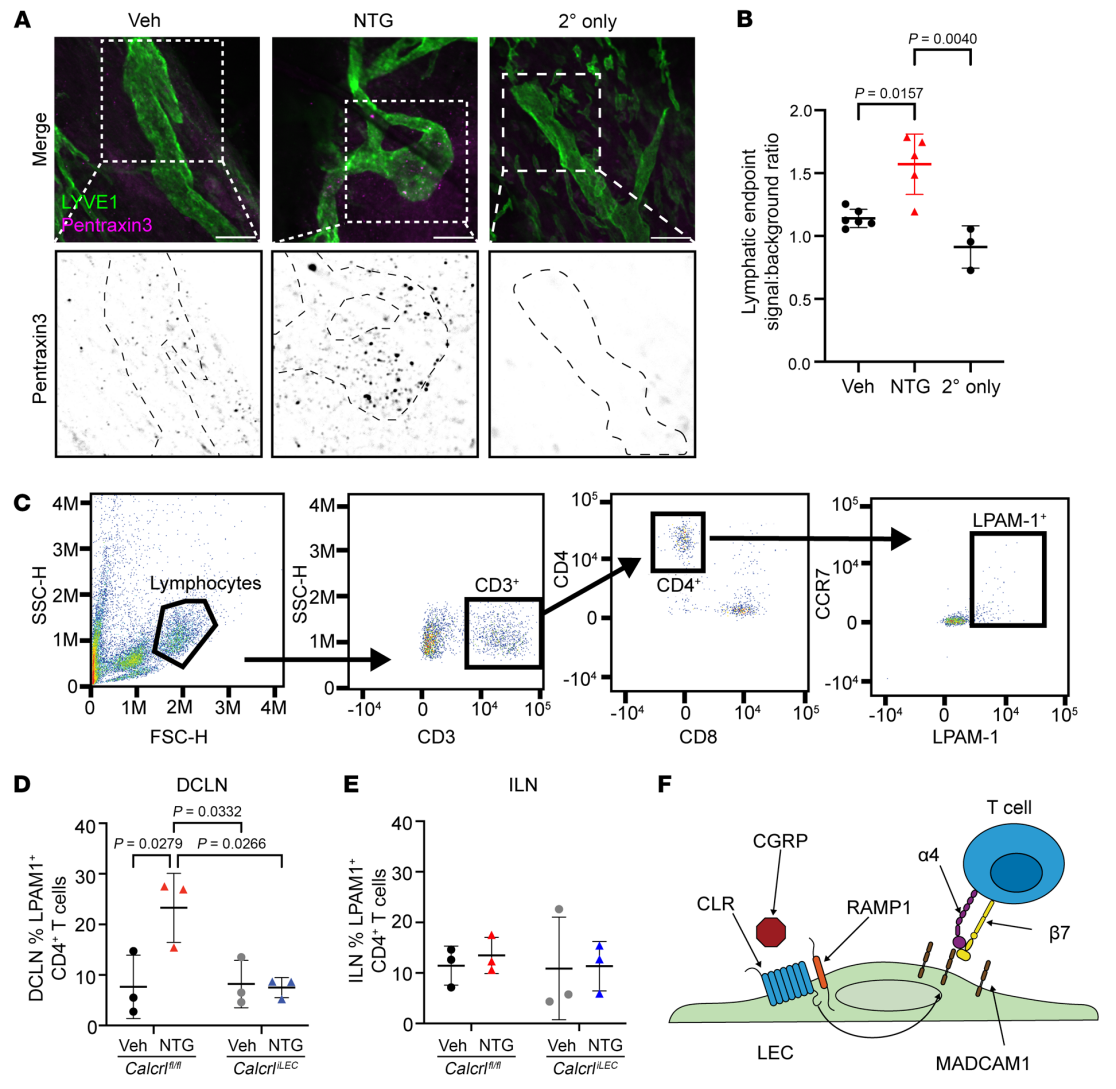
RiboTag and in vitro changes are recapitulated in NTG-mediated chronic migraine. (**A**) Whole-mount microscopy of decalcified meninges from mice treated with vehicle or NTG. Secondary-only immunofluorescence included as negative control. Top, costaining of LYVE-1 (green) and Pentraxin3 (magenta). Bottom, increased magnification images of the white dashed square in top row. Black, Pentraxin3. Black dashed lines, MLV outlines. Scale bar: 20 μm. (**B**) Quantification of PTX3 fluorescence relative to background fluorescence in MLV endpoints. Significance calculated using 1-way ANOVA with *n* = 3–6 animals with at least 2 endpoints assessed per animal. 2 independent cohorts were assessed. (**C**) Flow cytometry gating strategy. Quantification of flow cytometric analysis of LPAM1^+^ (α4/β7 integrin^+^) CD4^+^ T cells in (**D**) DCLNs (draining meninges) and (**E**) inguinal lymph nodes (distal lymph nodes) of NTG-treated chronic migraine *Calcrl^iLEC^* mice. *n* = 3 animals per group, pooled left and right DCLN from 2 independent cohorts, performed in duplicate. (**F**) Schematic indicating proposed relationship between CGRP, MADCAM1, and α4/β7 integrin^+^ (LPAM1^+^) CD4^+^ T cell interaction with LECs. Significance for all graphs calculated using 2-way ANOVA with Tukey’s multiple comparisons test. *P* value shown if less than 0.05. Graphs show mean ± SD.

MLVs have capillary endpoints that are hypothesized to be sites of CSF and immune cell egress from the CNS (3). Interestingly, *Ptx3* expression is increased at these blunt endpoints during macrophage-associated lymphatic malformation (62). To assess the expression of Ptx3 during chronic migraine, we performed whole mount immunofluorescence microscopy on decalcified dorsal skull with adherent meninges from vehicle or NTG-treated mice. Ptx3 protein expression was higher in the MLV capillary endpoints of NTG-treated mice compared with vehicle-treated mice (Figure 5, A and B). These Ptx3 positive endpoints may represent lymphatic vascular endothelial damage or priming of MLVs for immune cell interactions during chronic migraine.

Therefore, to assess whether there were differences in immune cell egress during NTG-induced chronic migraine, we performed multispectral flow cytometry of the DCLNs to which the MLVs drain (Figure 5C). Peripherally located inguinal lymph nodes (ILN), subjected to systemic NTG-injections but not implicated in chronic migraine pathophysiology, were included as controls. Because the RiboTag IPA analysis identified significant changes in both Th1 and Th2 responses, and because MADCAM1 is a potent adhesion molecule for α4/β7 integrin (LPAM1)–positive T cells, we elected to profile a broad range of T cell populations (see Supplemental Table 2). No differences were detected in Ifnγ^+^CD4^+^ Th1 cells, Gata3^+^CD4^+^ Th2 cells, CCR7^+^CD4^+^ T Cells, Ifnγ^+^CD8^+^ Tc1 cells, Gata3^+^CD8^+^ Tc2 cells, CCR7^+^CD8^+^ T Cells in DCLNs, nor in ILNs between NTG-treated or vehicle-treated *Calcrl^fl/fl^* and *Calcrl^iLEC^* mice (Supplemental Figure 3). However notably, the only significant change was an increase in the relative abundance of LPAM1 (α4/β7 integrin) positive CD4^+^ T cells in NTG-treated *Calcrl^fl/fl^* mice. This increase was not detected in NTG-treated *Calcrl^iLEC^* mice (Figure 5D), nor was it detected in peripheral ILNs (Figure 5E). α4/β7 integrin–positive CD4^+^ T cells are capable of interacting with endothelial cells expressing MADCAM1 (Figure 5F), which is significantly increased in lymphatics by CGRP stimulation (Figure 3E and Figure 4, G–I). These findings indicate that CGRP signaling in the lymphatic endothelium is required for the entry of LPAM1^+^CD4^+^ T cells into MADCAM1-enriched MLVs, leading to egress to the DCLN during chronic migraine (Figure 5F).

### CGRP induces formation of continuous, nonpermeable VE-cadherin junctions in cultured hLECs.

In addition to functioning as conduits for immune cell trafficking from peripheral tissues to secondary lymphoid organs, LVs maintain fluid homeostasis, including CSF turnover and efflux from the CNS through MLVs (1, 5, 8). Thus, changes in lymphatic endothelial cell junctions influence the permeability and function of LVs.

**Figure 6:**
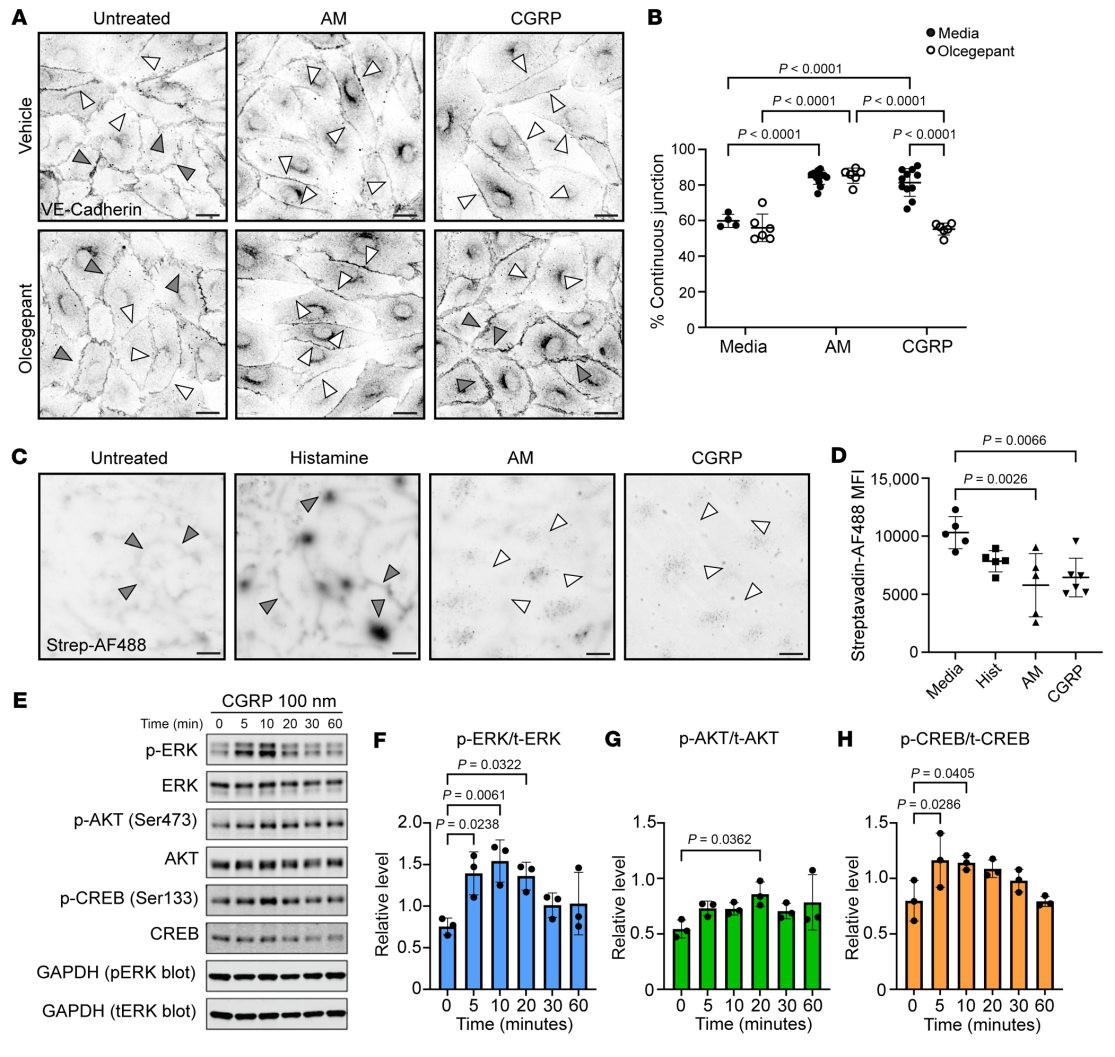
CGRP induces formation of continuous, nonpermeable VE-cadherin LEC junctions in cultured hLECs. (**A**) LECs treated with media, 100 nM adrenomedullin (AM) (low-permeability control), or 100 nM CGRP and treated with or without CGRP receptor antagonist olcegepant and incubated with antibodies targeting VE-cadherin. White arrows indicate continuous VE-cadherin arrangement, gray arrows indicate discontinuous VE-cadherin arrangement. Scale bar: 10 μm (**B**) Quantification of proportion of LEC continuous junctions treated with media, CGRP, or adrenomedullin. Significance calculated using 1-way ANOVA with Tukey’s multiple comparison’s test. *n* = 4–11 biological replicates with 2–3 randomly selected fields quantified per coverslip from 5 independent assays. All visible adherens junctions were counted and scored as either continuous or discontinuous by a blinded scorer. Percent continuous junctions is calculated as number of continuous VE-cadherin junctions divided by total number of observed junctions multiplied by 100. (**C**) Fluorescence microscopy of LECs grown on biotinylated-fibronectin coated coverslip and treated with 10 μM histamine (high permeability control), 100 nM adrenomedullin, or 100 nM CGRP or media alone then treated with Alexa Fluor-488–labeled streptavidin. Dark signal indicates increased Alexa Fluor-488 streptavidin permeability between LECs. White arrows indicate low permeability cell borders, gray arrows indicate highly permeable cell borders. Scale bar: 20 μm. (**D**) Permeability between LECs quantified as MFI of bound, labeled streptavidin. *n* = 5–6 biological replicates with 3–4 randomly selected fields quantified from 3 independent assays. MFI of each field assessed is averaged together to represent 1 biological replicate. Significance for **B** and **D** calculated using 1-way ANOVA with Tukey’s multiple comparisons test. Graphs = mean ± SD. (**E**) Representative Western blot assessing LEC intracellular signaling response to CGRP over 60 minutes. Quantification of phosphor-ERK (p-ERK) to total ERK (t-ERK) (**F**) p-AKT to t-AKT (**G**), and p-CREB to t-CREB (**H**) response to CGRP over 60 minutes. (**F**–**H**) significance was determined using an ordinary 1-way ANOVA with Dunnett’s multiple comparisons. 3 independent experiments were conducted.

To assess the role of CGRP on LEC junctions, hLECs were cultured in media or treated with 100 nM CGRP or 100 nM AM, a nonpermeabilizing agent, for 20 minutes with or without treatment with 3 nM olcegepant (a.k.a. BIBN4096), a small molecule receptor antagonist for CLR/Ramp1, which is the canonical CGRP receptor. The organization of interendothelial cell junctions was quantified using VE-cadherin staining and characterized as percent of total cell-cell junctional borders in the impermeable confirmation (Figure 6A). This measurement reflects a physiologic, protein-localization response, as it is assessed 20 minutes after treatment.

When compared with untreated cells the positive control AM peptide stimulated a robust increase in the linear organization of VE-cadherin (Figure 6A, white arrows), from 60% to approximately 85% continuous junctions (Figure 6B). As expected, olcegepant did not reverse the observed AM-mediated increase in linear junction because AM peptide signals through CLR/Ramp2 (Figure 6, B and C). Treatment with CGRP was equivalently potent in converting VE-cadherin arrangement to linear, continuous junctions (Figure 6A, white arrows), with a statistically significant increase to approximately 80% continuous junctions compared with untreated cells (Figure 6B). Treatment with 3 nM olcegepant was sufficient to prevent the CGRP mediated linearization of VE-cadherin junctional proteins, yielding a statistically significant reduction to approximately 60% continuous VE-cadherin junctions compared with CGRP treatment. There was no significant difference between CGRP-plus-olcegepant–treated LECs and olcegepant alone–treated LECs.

Further, the functional permeability of LEC monolayers to protein solutes was also assessed using similar treatment conditions. Confluent monolayers of hLECs were grown on biotinylated-fibronectin coated coverslips. Following 20 minutes of treatment conditions, fluorescent streptavidin (MW 60 kDa) was added to the cells for 3 minutes and the cells were fixed and stained for streptavidin. Permeability was quantified as mean fluorescence intensity (MFI) of streptavidin that passed between endothelial cell junctions (Figure 6C). Considering the inherent permeability of LEC junctions, the dynamic range of the assay is maximal under both untreated and histamine-treated conditions, with high streptavidin staining demarcating nearly all cell borders and abundant streptavidin puncta at multicellular junctions (Figure 6C, gray arrows). In contrast, MFI of hLECs treated with 100 nM CGRP was equivalent to the low permeability control of 100 nM AM, with a statistically significant reduction of 40% compared with media-alone conditions (Figure 6D). The very low levels of streptavidin staining between cell junctions (Figure 6C, white arrows) is consistent with the increased linear arrangement of VE-cadherin under CGRP and AM treatment conditions.

Protein quantification by Western blot illustrated ERK phosphorylation in cultured LECs as early as 5 minutes after treatment with CGRP persisting until 20 minutes after treatment and these changes were not observed in AKT phosphorylation (Figure 6, E–G). Similarly, CREB phosphorylation was detected as early as 5 minutes after CGRP treatment, persisting until 10 minutes after treatment (Figure 6, E and H). Taken together, increased VE-cadherin linearization and reduced protein transit through a monolayer indicated that CGRP structurally and functionally reduced LEC permeability in vitro, likely through ERK or CREB-mediated effects.

### NTG-induced CGRP signaling is required for formation of continuous VE-cadherin junctions in MLV capillary endpoints.

**Figure 7:**
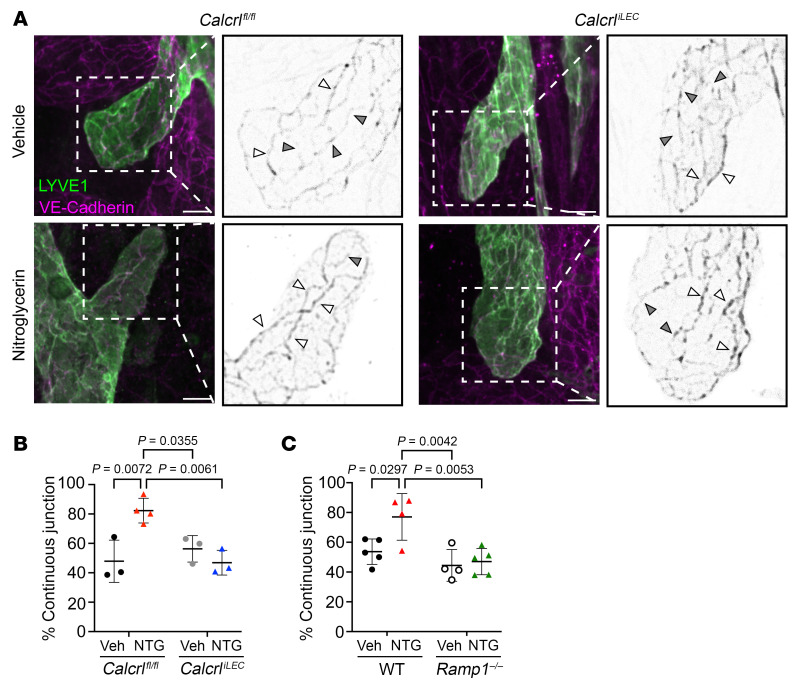
NTG induced CGRP stimulus is required for formation of continuous VE-cadherin junctions in vivo. (**A**) Whole-mount immunofluorescence microscopy targeting LYVE1-1 and VE-cadherin in NTG induced chronic migraine in *Calcrl^iLEC^* mice. White arrows, continuous VE-cadherin junctions, gray arrows, discontinuous VE-cadherin junctions. Scale bar: 10 μm. Black and white inset images are increased magnification of white dashed square. (**B**) Quantification of proportion of MLV endpoint linear VE-cadherin junctions in NTG-treated chronic migraine *Calcrl^iLEC^* mice. All visible VE-cadherin–positive adherens junctions were counted and scored as either continuous or discontinuous by a blinded scorer. Percent continuous junctions is calculated as number of continuous VE-cadherin junctions divided by total number of observed junctions multiplied by 100. *n* = 3–4 animals with percent continuous junctions scored from 2–5 meningeal LV endpoints assessed per animal from 2 independent cohorts. (**C**) Quantification of proportion of MLV endpoint linear VE-cadherin junctions in NTG-treated chronic migraine *Ramp1^–/–^* mice, calculated as in **B**. *n* = 4–5 animals per group scored from 2–5 meningeal lymphatic endpoints per animal from 3 independent cohorts. Graph shows mean ± SD. Significance for all graphs calculated using 2-way ANOVA with Tukey’s multiple comparisons test. *P* value shown if less than 0.05.

To determine whether endogenously elevated CGRP during chronic migraine affects the cellular junctions of MLVs in vivo, we quantified the arrangement of VE-cadherin within capillary endpoints of meningeal lymphatics of *Calcrl^fl/fl^* and *Calcrl^iLEC^* mice treated with vehicle or NTG, as described in Figure 1. All cell-cell junctions within 500 μm of lymphatic capillary end-points were assessed. The relative proportion of continuous VE-cadherin junctions (Figure 7A, white arrows) to discontinuous junctions (Figure 7A, gray arrows) in LYVE1-positive MLV endpoints was significantly increased to 80% in *Calcrl^fl/fl^* mice treated with NTG compared with vehicle-treated *Calcrl^fl/fl^* mice and vehicle-treated *Calcrl^iLEC^* mice (Figure 7B). Importantly, *Calcrl^iLEC^* mice treated with NTG had approximately 55% continuous VE-cadherin LEC junctions that were indistinguishable from the proportions observed in vehicle-treated *Calcrl^fl/fl^* and *Calcrl^iLEC^* animals (Figure 7, A and B).

Cell-cell junctions within 500 μm of lymphatic capillary end-points were also in systemically deficient *Ramp1^–/–^* mice. Vehicle-treated WT control animals exhibited similar phenotypes to *Calcrl^fl/fl^* mice (approximately 55% continuous junctions) as well as NTG-treated WT controls (approximately 75% continuous junctions) (Figure 7C). The proportion of continuous junctions in *Ramp1^–/–^* mice treated with vehicle was found to be approximately 45%, not significantly different from WT controls (Figure 7C). As expected, *Ramp1^–/–^* mice treated with NTG had approximately 50% continuous junctions, significantly less than WT NTG-treated animals and not significantly different from *Ramp1^–/–^* mice treated with vehicle (Figure 7C). Taken together, these data indicate that chronic migraine–associated rearrangement of MLV VE-cadherin cell junctions requires CGRP signaling.

### CGRP reduces MLV permeability and CSF drainage to DCLN in vivo.

To directly test the effect of CGRP on meningeal lymphatic vascular permeability in vivo, 5 μL of 1% Evans blue (EB) in normal saline with or without 5 μg CGRP ([CGRP] = 1 μg/μL) was injected into the cisterna magna of *Calcrl^fl/fl^* and *Calcrl^iLEC^* mice. EB is a traceable dye that can be detected colormetrically by light microscopy or fluorescently after bound to albumin by fluorescent emission in the red and far-red wavelengths. Thus, ICM injections of EB can be utilized to quantify CSF uptake by MLVs and relative efflux of CSF to the DCLNs (8).

**Figure 8:**
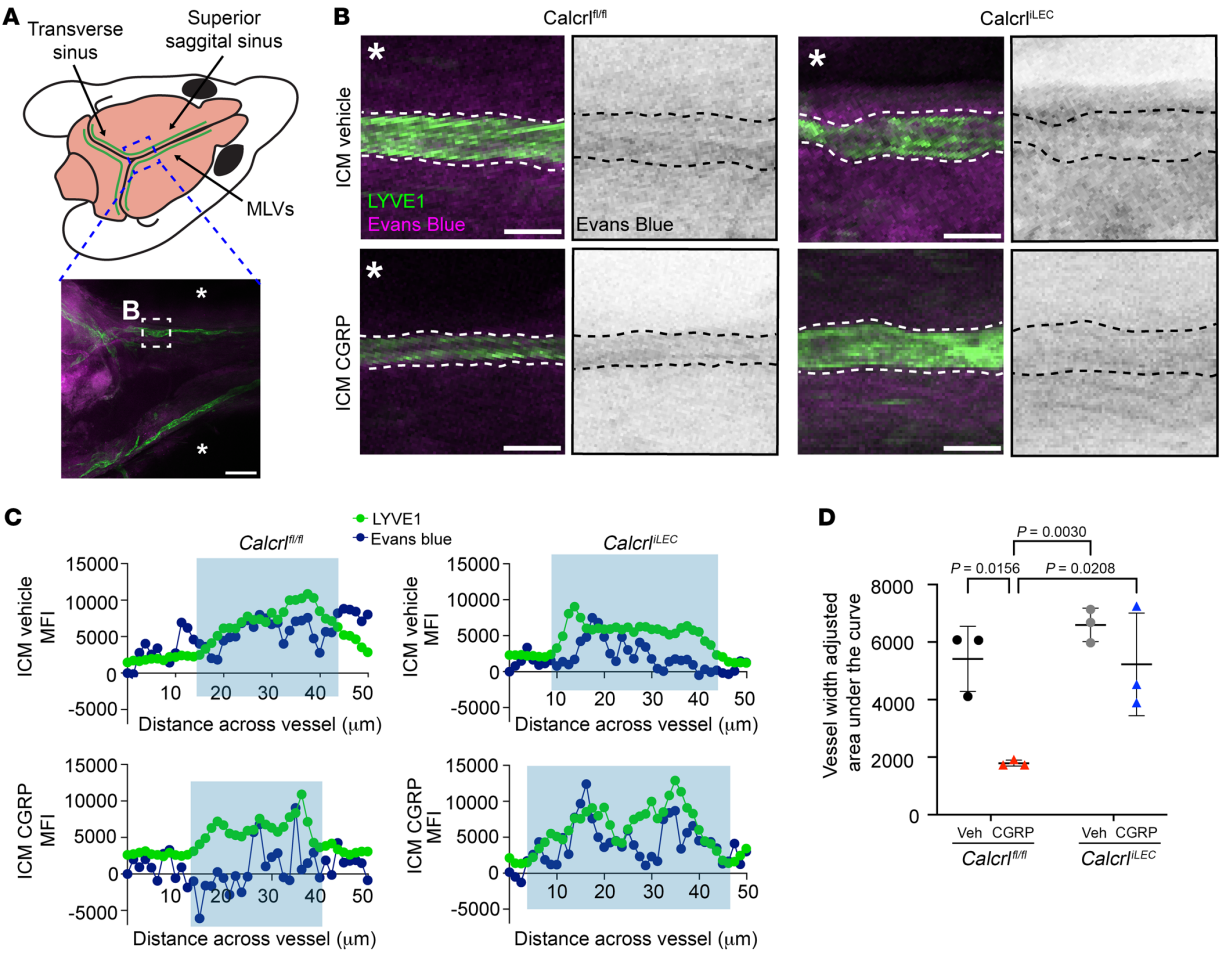
CGRP reduces CSF uptake into MLVs. (**A**) Schematic of MLV anatomy. Blue dashed square indicates location assessed, while the white dashed square indicates the location for dye measurement, as visualized in **B**. The asterisk (*) indicates the space outside the sinus. Scale bar: 200 μm. (**B**) Left, representative image of LYVE1+ (Green) MLVs outlined with white dashed line. Right, EB fluorescence in black. Black dashed line, MLV border. Scale bar: 50 μm. (**C**) Representative integrated fluorescence intensity across MLV measured using plot profile feature in ImageJ. An average background fluorescence intensity for each field was measured and subtracted from raw EB fluorescence intensity values. Green line, LYVE1 MFI, Blue line, EB MFI, Blue box, margins of MLV. (**D**) Quantification of background adjusted integral of EB intensity across MLV. *n* = 3 animals 1 drainage site assessed per animal from 3 independent cohorts. Graph shows mean ± SD. Significance calculated using 2-way ANOVA with Tukey’s multiple comparisons test. *P* value shown if less than 0.05.

EB solution diluted in normal saline was readily observed within the LYVE1^+^ LVs of the superior sagittal sinus of both *Calcrl^fl/fl^* and *Calcrl^iLEC^* mice, visualized by confocal and epifluorescence microscopy of decalcified meninges (Figure 8, A and B). Using fluorescence intensity plot profiles across the vessel, the area under the EB curve between the borders of the LYVE1^+^ MLVs was calculated and adjusted to vessel width for an overall quantitation of MLV permeability (Figure 8, C and D). With this quantitation method, there was no significant difference in MLV permeability or dye uptake between vehicle-injected *Calcrl^fl/fl^* and *Calcrl^iLEC^* animals (Figure 8, B–D). However, inclusion of CGRP in the EB injection caused a marked and significant decline in MLV dye uptake in *Calcrl^fl/fl^* mice (Figure 8, B–D), consistent with the robust nonpermeabilizing effects of CGRP observed in vitro and in vivo (Figures 5 and 6). As expected, *Calcrl^iLEC^* mice with loss of the CGRP receptor in lymphatics were unresponsive to CGRP/EB injection and did not differ significantly from vehicle-treated *Calcrl^fl/fl^* and *Calcrl^iLEC^* animals.

**Figure 9:**
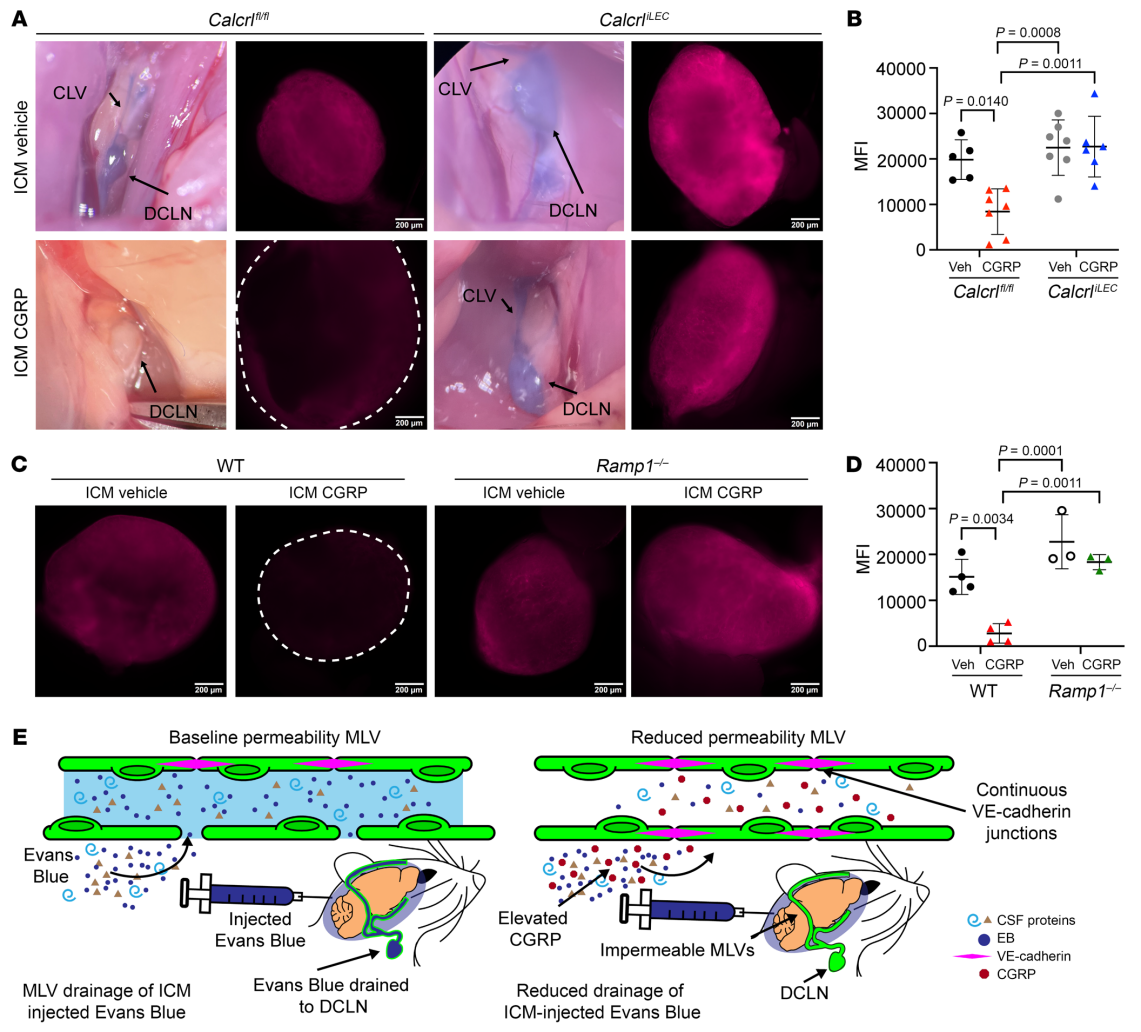
ICM injection of CGRP reduces CSF efflux to the DCLNs by EB dye transport. (**A**) Photograph and whole mount fluorescence microscopy of DCLN from *Calcrl^iLEC^* mice injected intra cisterna magna (ICM) with 1 μg/μL CGRP or vehicle diluted in 1% EB. Scale bar: 200 μm. CLV, cervical LV, DCLN, deep cervical lymph node. (**B**) Quantification of MFI of DCLN of *Calcrl^iLEC^* mice injected ICM with 1 μg/μL CGRP or vehicle diluted in 1% EB. *n* = 5–7 animals, average MFI of left and right DCLNs graphed from 4 independent cohorts. Graph shows mean ± SD. Significance calculated using 2-way ANOVA with Tukey’s multiple comparisons test. *P* value shown if less than 0.05. (**C**) Whole mount fluorescence microscopy of DCLN from *Ramp1^–/–^* or WT mice injected intra cisterna magna (ICM) with 1 μg/μL CGRP or vehicle diluted in 1% EB. Scale bar: 200 μm. (**D**) Quantification of MFI of DCLN of *Ramp1^–/–^* or WT mice injected ICM with 1 μg/μL CGRP or vehicle diluted in 1% EB. *n* = 3–4 animals, average MFI of left and right DCLNs graphed from 3 independent cohorts. Graph shows mean ± SD. Significance for calculated using 2-way ANOVA with Tukey’s multiple comparisons test. *P* value shown if less than 0.05. (**E**) Representative schematic of CGRP impact on MLVs demonstrating reduced MLV permeability and CSF efflux to the DCLN.

As a secondary measure of CSF efflux, EB transit from the MLVs to the draining DCLNs located below the sternocleidomastoid muscle was quantified. DCLNs were visualized and dissected bilaterally (Figure 9A) and MFI of bilateral DCLNs was recorded using epifluorescence microscopy (Figure 9A) and averaged to represent net MLV drainage by animal (Figure 9B). Robust amounts of EB dye were visually present and quantified by epifluorescence in the DCLNs of both *Calcrl^fl/fl^* and *Calcrl^iLEC^* mice injected with vehicle diluted in EB 5 minutes after ICM injection (Figure 9, A and B). Remarkably, inclusion of CGRP in the EB dye robustly reduced EB MFI in the DCLNs of *Calcrl^fl/fl^* mice, with no visible EB dye present in the DCLNs and with dramatically reduced DCLN fluorescence in the red channel 5 minutes after ICM injection (Figure 9, A and B). This attenuation of MLV drainage to the DCLN was specific to CGRP peptide and EB fluorescence in *Calcrl^iLEC^* mice injected ICM with CGRP/EB did not differ significantly from vehicle-treated *Calcrl^fl/fl^* and *Calcrl^iLEC^* animals.

CGRP-mediated reduction of EB dye was also assessed in systemically deficient *Ramp1^–/–^* mice. Consistent with *Calcrl^fl/fl^* mice, WT control mice demonstrated robust EB fluorescence in DCLNs bilaterally and reduction in EB fluorescence with CGRP coinjection (Figure 9, C and D). As expected, *Ramp1^–/–^* mice exhibited EB fluorescence consistent with WT baseline (Figure 9D). Inclusion of CGRP in the EB dye injected in *Ramp1^–/–^* mice did not affect EB fluorescence in the DCLNs, again suggesting that CGRP signaling through CLR/Ramp1 is sufficient to change CSF efflux through the meningeal lymphatic vasculature. Collectively, these data demonstrate that lymphatic-CGRP signaling through CLR/Ramp1 markedly reduced CSF outflow to the DCLNs by rearranging lymphatic VE-cadherin endothelial junctions and thereby reducing MLV permeability (Figure 9E).

## Discussion

Despite its notoriety as the third most prevalent illness globally, affecting more than 10% of the worldwide population (10, 65), the etiology and pathophysiology of migraine remains poorly understood. Here, we used a well-established murine model of chronic migraine coupled with 2 genetic models of CGRP signaling insufficiency, *Ramp1* systemic deficiency and lymphatic-specific *Calcrl* insufficiency, to discover a previously unrecognized role of meningeal lymphatics in chronic migraine pain and pathophysiology. Loss of CGRP signaling in lymphatics significantly ameliorated chronic migraine pain in mice, whereas direct injection of CGRP into the cisterna magna reduced MLV permeability and abrogated CSF drainage. These findings were consistent with our findings in Ramp1 systemically deficient animals that have no tissues capable of robustly responding to CGRP. Overall, these data support that elevated levels of CGRP during chronic migraine can act on MLVs to reduce their permeability and CSF drainage functions. This reduced permeability may culminate in an increase in CSF pressure (66, 67) or in the retention of vascular and immune promigraine factors within the dura (68), exacerbating the propagation of chronic migraine pain.

Monoclonal antibodies targeting either the CGRP receptor (erenumab) or plasma CGRP (fremanezumab, galcanezumab, epitinizumab), and small molecule antagonists of the CGRP heteroreceptor, CLR/RAMP1, are effective antimigraine therapeutics (25, 26). However, the cellular targets of these medications remain unknown (21). I-125–labeled galcanezumab has been measured in meningeal tissue at approximately 10% of its plasma concentration (69), and fluorescently labeled fremanezumab was detected in the dura, dural blood vessels, and trigeminal ganglion, but not in the central nervous system (70). These data support the plausibility that MLVs represent a previously unappreciated CGRP effector target within the trigeminovascular system. Although considered revolutionary, only 50% of patients on the highest dose of erenumab experience a greater-than 50% reduction in monthly migraine days (71). This partial reduction of chronic migraine symptoms correlates with our observations of approximately 50% attenuation of migraine symptoms in mice with impaired lymphatic response to CGRP compared with the total prevention, as observed in *Ramp1^–/–^* mice. Because our data demonstrates a full protection in systemic Ramp1-deficient animals and partial protection in lymphatic *Calcrl-*deficient animals, our findings indicate that migraine pathophysiology is multifactorial and that the MLVs are one of many CGRP target cells.

Reduced meningeal lymphatic and glymphatic CSF efflux has so far been observed in patients with spontaneous chronic migraine by MRI, and reduced flow correlated negatively with worsened migraine symptoms (72). These changes were observed both during and between migraine episodes. Altered CSF efflux through the MLVs has been implicated in a variety of additional neurodegenerative and neuroinflammatory diseases, including Alzheimer’s disease (AD), Parkinson’s disease, multiple sclerosis, traumatic brain injury, brain tumors, and also in aging. Disruption of CSF efflux through MLVs caused increased amyloid-β deposition in the meninges and increased perivascular plaque formation in an AD mouse model (3). Interestingly, the incidence of AD in patients with migraine is nearly twice the rate of AD in people who were healthy controls (73). Additionally, α-synuclein pathology correlates with delayed MLV drainage, and blocked MLV flow exacerbates motor and memory deficits in a mouse model of Parkinson’s disease (6). Moreover, increased CSF pressures are causally associated with another chronic headache disorder, idiopathic intercranial hypertension, and reduction of elevated CSF pressure alleviates pain (74). If, as suggested by our data, chronic migraine–induced CGRP triggers transient changes in CSF efflux, then these acute changes may propagate migraine pain, while chronic alterations to CSF efflux over decades of migraine episodes may permanently damage the MLVs, potentially contributing to the pathogenesis or pathophysiology of Alzheimer’s dementia other neurodegenerative or neuroinflammatory conditions. Promisingly, our data strongly suggests that CGRP inhibitor therapeutics may act to restore MLV drainage during a migraine attack, which is important for the prevention of long-term CSF efflux–related diseases.

We leveraged lymphatic-specific RiboTag mice to develop a deeper mechanistic understanding of how the cellular transcription of meningeal LECs changes in response to migraine. We identified 3 promising translationally altered immunovascular proteins associated with chronic migraine and CGRP signaling: *GJC2*, *PTX3*, and *MADCAM1*.

*GJC2* encodes for connexin-47 (Cx47) and is a causally mutated in some forms of human primary lymphedema (60). Cx47 forms homotypic gap junctions as well as heterotypic gap junctions with Cx43, which is also causally associated with lymphedema in humans (75). Using cultured hLECs, we noticed that CGRP promoted the localization of Cx47 along continuous, low permeability VE-cadherin adherens junctions. Interestingly, tonabersat, a Cx43 gap junction signaling inhibitor is an effective prophylaxis for migraine attacks with aura, though it was found to be ineffective for migraine attacks without aura (76). These data suggest that under migraine conditions of elevated CGRP, lymphatics may exhibit increased intercellular gap junction communication that may be therapeutically targeted.

*PTX3* is a member of the pentraxin family and, alongside C-reactive peptide, is an acute phase reactant in the humoral immune response. PTX3, which is expressed by LECs, is a newly characterized serum biomarker of migraine attacks (56–58). Additionally, Ptx3 defines a novel subset of immune-interacting, capillary LECs that promote pathologic LV remodeling (62). Our current results suggest that PTX3 upregulation in MLV endpoints during migraine may indicate a state of endothelial damage and/or function as a chemoattractant for egressing immune cells.

MADCAM1 typically interacts with α4β7 integrins to recruit immune cells to mucosal tissues such as the gut, but it is also expressed by some LECs (77–79). Likely correlated with the upregulation of *Madcam* expression by CGRP, we also discovered a significant increase in the relative abundance of CD4^+^ T cells expressing LPAM1 (α4β7 integrin) in the DCLNs of mice with NTG-induced migraine. These LPAM1^+^ (α4β7 integrin) CD4^+^ T cells are critical for the initiation and development of inflammatory bowel disease (IBD) (80, 81) and are being targeted by monoclonal antibodies etrolizumab (β7) and natalizumab (α4). While the current study is the first to implicate these T cells with migraine, it may be worth noting that patients with IBD have an increased prevalence of migraine headache compared with the general population (82, 83), hinting at a potential link between CGRP-mediated gut immunity and migraine.

Taken together, this work provides the first demonstration of the importance of the lymphatic vascular system in the pathophysiology of migraine along with several plausible mechanistic frameworks for understanding the complex CGRP-mediated neurological, vascular, and immune interactions in chronic migraine pathophysiology. The impact of migraine-related reductions of CSF efflux and of potential chronic MLV dysfunction on the development of neurodegenerative diseases remains to be fully characterized. Future studies to evaluate the contributions of MLV CSF drainage in humans during migraine, with and without CGRP-targeted therapies, is warranted.

## Methods

Additional Experimental Details are provided in Supplemental Methods.

### Sex as a biological variable.

Female and male *C57BL/6J*, and *Calcrl^iLEC^* mice aged 3–6 months were used to characterize the role of lymphatic CGRP signaling in chronic migraine. Female mice were used for all facial expression of pain and light avoidance behavioral assays. This study primarily used female mice because the human disease condition is predominant in females, with 3–5 times higher incidence in women than men.

### Animals.

*C57BL/6J* mice were obtained from Jackson Laboratories or bred in house. *Calcrl^fl/fl^* mice were generated as previously described (38). *Vegfr3-Cre^ERT2^* mice were also generated and described previously (47), as were *Ramp1^–/–^* mice (84). To establish deletion of *Calcrl*, *Calcrl^fl/fl^*;*Vegfr3^CreERT2^* (*Calcrl^iLEC^*) and control *Calcrl^fl/fl^*, mice were injected intraperitoneally with 100 μg/g body weight tamoxifen (T5648, Sigma-Aldrich) diluted in corn oil for 5 consecutive days. Experimental protocols were initiated 1 week after the termination of tamoxifen administration. Deletion of *Calcrl* was determined by qPCR of mRNA isolated from immunoprecipitated LECs from meninges and nonmeningeal tissue. For translatomic analysis, *C57BL/6J* mice were obtained from Jackson Labs. For translational analysis, *B6J.129(Cg)-Rpl22^tm1.1Psam^/J* (RiboTag) mice were obtained from Jackson Labs. All mice were bred and maintained in a specific pathogen–free facility at the University of North Carolina at Chapel Hill School of Medicine, Chapel Hill, North Carolina.

### Lymphatic endothelial cell immunoprecipitation from tissue.

Single-cell suspension of meningeal tissue was negatively selected with CD68 immunoprecipitation and positively selected with Lyve1^+^ immunoprecipitation to generate enriched LECs.

### NTG chronic migraine model.

Mice were injected intraperitoneally (IP) with 10 mg/Kg body weight of sterile NTG (T-021, Sigma-Aldrich) diluted in 0.9% saline (PAA128035, Hospira) with vehicle consisting of 0.9% normal saline containing 10% propylene glycol or with 0.9% normal saline every other day for up to 5 injections. Vehicle injections consisting of 0.9% normal saline with 10% propylene glycol (the solvent for commercially available NTG) did not provoke significantly different pain behavior compared with 0.9% normal saline alone injections (data not shown).

Pain assessment was performed using the murine grimace scale, as previously described (48, 85), and using light aversion and movement behavior, as described in Supplemental Methods.

### ICM injections and CSF drainage quantification.

Animals were anesthetized using 20 μL/10 g body weight of 1.25% avertin (T48402, Sigma-Aldrich). After a sagittal skin incision, muscle layers were retracted and 5 μL of 0.9% saline containing 1 μg/μL CGRP (015-02, Phoenix Pharmaceuticals) and 1% Evans Blue (E2129, Sigma-Aldrich) or 1% Evans Blue vehicle control solution was injected ICM over 60 seconds. The needle was removed after 5 minutes. Deep cervical lymph nodes, inguinal lymph nodes, and cranial meninges were dissected 2 minutes following retraction of needle. Collected tissues were fixed in 4% paraformaldehyde for up to 24 hours. Lymph nodes were imaged using an I83 Olympus inverted fluorescence microscope with 10 × objective lens, connected to a Hamamatsu camera. Mean fluorescence index was quantified using ImageJ (86).

### Whole mount immunofluorescence.

Dorsal skull bones with adherent meninges were fixed in 4% paraformaldehyde for 24 hours and decalcified using 0.5 M EDTA (02793, Thermo Fisher Scientific) in PBS for 5–7 days at 4°C on a rocking platform in the dark. Tissues were blocked and permeabilized in PBS containing 5% normal donkey serum (017-000-121, Jackson ImmunoResearch), and 0.5% Tween-20 (BP337 Thermo Fisher Scientific) for 24 hours at 4°C and then incubated with antibodies detailed in the immunofluorescence antibody table at the indicated dilution for 24–48 hours at 4°C and then washed 3 times in PBS containing 5% normal donkey serum and 0.5% Tween-20. Secondary antibodies were diluted in PBS containing 5% normal donkey serum for 4 hours at room temperature or 24 hours at 4°C. Decalcified dorsal skull and meninges were mounted in 50% glycerol (BP2291, Thermo Fisher Scientific), and imaging was performed using a Zeiss 800 upright confocal microscope.

### Flow cytometry.

Multispectral flow cytometry was performed on single-cell suspensions of bilateral DCLNs or inguinal lymph nodes, as further described in the Supplemental Methods.

### LEC culture and analyses.

LECs (c-12217, PromoCell Inc.) were cultured in endothelial supplemental media MV2 (C-22121, PromoCell Inc.) to 3–5 passages from primary sample collection and were grown at 37°C with 5% CO_2_. Confluent monolayers of LECs were used for immunocytochemistry, permeability assays, and Western blot analysis, as described in Supplemental Methods.

### RiboTag gene expression analysis.

*Rpl22^HA/+^;Lyve1^Cre^* (52) animals were induced with chronic migraine following the protocol listed above. Sixty minutes after the last injection, animals were euthanized using CO_2_ asphyxiation. After preparation of the dorsal skull bone, as described above, cranial meninges were dissected using fine-tipped forceps and the whole meningeal tissue was lysed using Lysing Matrix D beads (116913100, MP Biomedicals) in 50 mM Tris, 10 mM KCl (51343180, Mettler-Toledo), 12 mM MgCl_2_ (AM9530G, Thermo Fisher Scientific), and 1% nonidet-p40 (198596, MP Biomedical) “Homogenization Buffer”. Hemagglutinin-tagged ribosomes were immunoprecipitated using Pierce anti-HA–conjugated magnetic beads (88837, Thermo Fisher Scientific) in supplemented homogenization buffer containing the above reagents with 0.5 mM DTT (7016L, Cell Signaling), 100 μg/mL cycloheximide (01810, Sigma-Aldrich), 1 mg/mL Heparin (BP2524100, Thermo Scientific), 200 units/mL RNase out (10-777-019, Thermo Fisher Scientific), and 1.5 × Protease inhibitor cocktail (11697498001, Sigma-Aldrich). Anti-HA beads were incubated with lysed meninges samples for 24 hours on a rotator at 4°C. HA-Tagged ribosomes were eluted from beads using a modified homogenization buffer with 300 mM KCl. RNA was isolated using RNeasy Kits (13997, Qiagen). Clariom S Pico microarray targeting mouse (902932, Thermo Fisher Scientific) was used in collaboration with the UNC Functional Genomics Core to measure expression changes. Data analysis was completed using Partek Genomics Suite (Partek) and Ingenuity Pathway Analysis (Qiagen) software programs.

### Statistics.

Data were analyzed using either 2-tailed Student’s *t* test with or without Welch’s correction, 1-way ANOVA with multiple comparisons and Tukey’s post hoc test, or 2-way ANOVA with multiple comparisons and Tukey’s post hoc test. The statistical test performed is noted in corresponding figure legends. *P* values under 0.05 were considered significant, and precise *P* value is depicted on figures where appropriate. The persons performing the analyses were blinded to the genotype of treatment of the animals or cells until the end of analysis.

### Study approval.

All protocols involving animals were approved by the UNC-CH’s IACUC.

### Data availability.

Microarray data is provided in the GEO database under accession number GSE266558. All data in the manuscript is included in the Supporting Data Values file.

### Online supplemental material.

Expanded methods are available in the supplemental material. Supplemental Table 1 provides raw data for all genes queried using the Clariom S microarray. Supplemental Table 2 provides a list of antibodies used for immunostaining and for flow cytometry.

## Author contributions

NPNM designed research studies, acquired and analyzed data, prepared figures, wrote and edited the manuscript, and acquired funding. LB conducted experiments, acquired and analyzed data, and edited the manuscript. ALSB conducted experiments and acquired and analyzed data. BMK acquired data, analyzed blinded behavioral data and analyzed data. SDS performed western blot and acquired data. ESD analyzed data. AHS conducted experiments and acquired data. AMT conducted experiments and acquired data. KMC designed research studies, supervised the research, acquired funding, and wrote, and edited the manuscript.

## Supplementary Material

Supplemental data

Unedited blot and gel images

Supplemental table 1

Supporting data values

## Version 1 05/14/2024

In-Press Preview

## Version 2 08/01/2024

Electronic publication
