# Supplementary material for: Meningeal lymphatic CGRP signaling governs pain via cerebrospinal fluid efflux and neuroinflammation in migraine models
Source: J Clin Invest. 2024 May 14;134(15):e175616. doi: 10.1172/JCI175616 (PMC11290972; doi:10.1172/JCI175616)
Supplement: Supplemental data [file jci-134-175616-s220.pdf]

Supplemental Materials

Supplementary Methods:

Lymphatic endothelial cell immunoprecipitation from tissue

Meningeal tissue was dissected and incubated for 15 minutes in 2 mg/mL collagenase type II (Worthington Biochemical 4176) diluted in Hank's Balanced Saline Solution in a 37 °C water bath. Single cell suspensions were passed through a 70 µm filter and centrifuged in an Eppendorf centrifuge 5810R at 1200 RPM for 5 minutes. Pellets were incubated with CD68 monoclonal antibody (Bio-Rad MCA1957) adsorbed Protein G Dynabeads (Thermo Fisher Scientific 10004D) in 1% BSA in PBS for 1 hour at 4 °C on a rotator. CD68+ cells were immunoprecipitated and washed three times with 1% Bovine Serum Albumin (BSA) in PBS. The negative fraction was saved and centrifuged at 300 Relative Centrifugal Force (RCF) in a Denville 260D centrifuge for 10 minutes. Cell pellets were incubated with Lyve-1 monoclonal antibody (Fitzgerald 70R-LR005) in 1% BSA in P0BS for 1 hour at 4 °C on a rotator. Lyve-1 adsorbed Dynabeads were immunoprecipitated using a magnet and were washed three times with 1% BSA in PBS. The Lyve-1 positive Dynabead positive portion was centrifuged at 300 RCF in a Denville 260D centrifuge for 10 minutes and the supernatant was discarded. The cell pellet was lysed in 600 uL RLT buffer (From Qiagen RNEasy kit) and frozen to -80 °C. RNA was isolated using an RNEasy kit (Qiagen 74104).

Nitroglycerin migraine model

Mice were injected intraperitoneally (IP) with 10 mg/Kg body weight of sterile nitroglycerin (T-021, Sigma-Aldrich) diluted in 0.9% saline (PAA128035, Hospira) with vehicle

consisting of 0.9% normal saline containing 10% propylene glycol or with 0.9% normal saline every other day for up to 5 injections. Vehicle injections consisting of 0.9% normal saline with 10% propylene glycol (the solvent for commercially available NTG) did not provoke significantly different pain behavior compared to 0.9% normal saline alone injections (data not shown).

Behavioral assays were performed 30 minutes after injection of nitroglycerin on each test day (days numbered day 1, 3, 5, 7, or 9 in figures). Facial expression of pain (murine grimace scale) was recorded for 20 minutes using a video camera to assess facial expression of pain. Pain response was assessed from 30 minutes to 50 minutes post NTG injection. To generate murine grimace scores, still images were captured every 60 +/- 10 seconds for the full length of the video. Images were scored manually by a blinded reviewer using the Murine Grimace Scale. The sum of each scored category for each frame were then averaged together to generate an average score for the whole test period. Four independent experimental cohorts were assessed for *Calcrl*^{iLEC} experiments and three independent experimental cohorts were assessed for *Ramp1*$^{-/-}$ experiments. Experiments were conducted at room temperature (22 °C).

Prior to light aversion testing, animals were allowed to acclimate to the testing chamber for 30 minutes 4-5 days before the first migraine induction day. Baseline measurements were recorded two days before the first migraine induction day, noted as day -1. On migraine induction days (noted as day 1, 3, 5, and 7) mice were allowed to acclimate to the room with all of the equipment turned on for 30 minutes in their home cage prior to IP injection with NTG or saline to initiate the experimental model. After IP injection of NTG or of saline, mice were allowed to rest for 30 minutes in their home cages. Then, light aversion behavior was recorded for 30 minutes using a VersaMax Legacy Open Field – Locomotor Activity system with an

inserted dark chamber constructed from black plexiglass. The light was generated by LED lamps with a maximum intensity of 1000 lumens. Cage temperatures did not rise above 25°C. Twelve independent experimental cohorts were assessed over the course of 2 years. Animals were given ad libidum access to food and water throughout testing, except for 30 minutes while in the VersaMAX testing chamber. Percent time moving while in the dark chamber was calculated as time moving in dark chamber divided by total time in dark chamber multiplied by 100. This measurement was normalized to the baseline measurement measured on day -1 and calculated as % time moving dark experimental day divided by % time moving in dark chamber on second baseline day multiplied by 100.

Flow cytometry

Bilateral deep cervical or inguinal lymph nodes were ground through 40 µm filters and rinsed with 5 mL of PBS supplemented with 1% Bovine Serum Albumin (FACS buffer) (BSA, BP1600, Fisher Scientific). All following steps were performed on ice or at 4°C. Cells were incubated with Brefereldin A (eBioscience, 00-4506-51) for 30 min. Cells were washed in FACS buffer and centrifuged at 450 RCF for 15 min. FC receptors were then blocked with FcR block (Milteny Biotec 120-003-855) for 15 minutes with 1 uL per 1,000,000 cells. Cells were then washed in FACS buffer and centrifuged at 450 RCF for 15 minutes. Surface antigens were stained for 30 minutes with antibodies as listed in table S2. Cells were washed in FACS buffer and centrifuged at 450 RCF for 15 minutes, and then incubated with Fix and Perm buffer (BD Biosciences 554714) for 20 min and washed in Perm/wash buffer from the kit and centrifuged at 450 RCF for 15 minutes. Cells were then stained with antibodies for intracellular antibodies and

live/dead marker as listed in table S2. Data were collected during the same day as dissection and acquired using a Cytek Aurora multispectral flow cytometer (Cytek Biosciences). Analysis was performed using FlowJo software (TreeStar).

Fluorescent immunocytochemistry

Lymphatic endothelial cells (LECs) (c-12217, PromoCell Inc.) were cultured in endothelial supplemental media MV2 (C-22121, PromoCell Inc.) to 3-5 passages from primary sample collection and were grown at 37°C with 5% CO_2. LECs were treated either with 100 nM CGRP (015-02, Phoenix Pharmaceuticals), 100 nM adrenomedullin (4030315.1000, Bachem), or media with or without olcegepant (also referred to as BIBN 4096, Tocris 4561) in endothelial supplemental media MV2 (PromoCell Inc. C-22022) or untreated media for 20 minutes for junctional arrangement or 8 hours for RNA analysis by qPCR. Cells were fixed in 4% paraformaldehyde for 20 minutes at RT, followed by incubation with PBS containing 5% normal donkey serum overnight at 4°C. Samples were then incubated with antibodies detailed in the immunofluorescence antibody table at the indicated dilution in PBS 5% NDS for 1 hour, and then gently washed with PBS. Secondary antibodies were diluted as detailed in the immunofluorescence antibody table at the indicated dilution in PBS containing 5% normal donkey serum for 2 hours at RT. Cells were incubated with HOECHST nuclear stain at 1:1000 for 10 minutes then washed in PBS and mounted in ProLong Gold antifade media (P36934, Life Technologies). Cells were imaged using a Zeiss 800 confocal microscope and an I83 Olympus inverted fluorescence microscope with Hamamatsu camera.

LEC permeability assay

LECs were grown to confluency on coverslips coated with EZ-link sulfo-NHS-LC-biotin (A39257, Thermo Scientific) conjugated fibronectin (PHE0023, Gibco). Cells were treated with either 100 nM CGRP, 100 nM adrenomedullin, 10 µM histamine, or untreated media for 20 minutes and then treated with 25 µg/mL AF488 conjugated streptavidin (S32354, Thermo Fisher Scientific) for 3 minutes. Cells were fixed with 4% paraformaldehyde for 20 minutes and mounted in ProLong Gold antifade media. Cells were imaged using a Zeiss 800 confocal microscope and an I83 Olympus inverted fluorescence microscope with Hamamatsu camera.

Western Blot

LECs were seeded at 1.2 x 10^5 cells per well in a 6-well plate. Once cells were confluent, media was replaced with Opti-MEM (Gibco, #31985070) to serum-starve cells overnight. The following day, cells were treated with 100 nM CGRP for a time-course from 0 to 60 minutes. At the end of the time-course, the media was replaced with ice cold 1x dPBS to stop signaling. The solution was aspirated and remaining cells were lysed in 50 mM Tris-HCL (pH 7.4), 150 mM NaCl, 1% NP-40, 0.25% deoxycholate, 1 mM EDTA, 2 M urea) supplemented with protease (ROCHE, #11873580001) and phosphatase (ROCHE, #4906845001) inhibitors and Benzonase® nuclease (Millipore, #E1014). Protein was measured and normalized using a BCA assay kit (ThermoFisher Scientific, #23225) and 4x LDS sample buffer (Invitrogen, #NP0007) supplemented with 10% DTT. Samples were boiled for 5-10 minutes at 95ºC and run on a 4-12% NuPAGE™ Bis Tris gel (ThermoFisher Scientific, #NPO336BOX) using 1x MOPS-SDS Running buffer (Boston BioProducts, #BP-178). Gel separated proteins were transferred to a nitrocellulose membrane using 2x Transfer Buffer (Boston BioProducts, #BP-193) supplemented with 20% methanol. Membranes were blocked in 1xTBS/T + 5% BSA. Antibody-bound membranes were imaged on a

Licor Odyssey CLx using Image Studio version 5.2. Densitometry was performed using ImageJ. Two gels were run for each sample; one to probe for phosphorylation of ERK, AKT, and CREB and the other to probe for total levels, respectively. GAPDH served as a load control. Protein quantification of each sample was determined by the following: [(phosphorylation protein level/GAPDH) / (Total protein level/GAPDH)]. Antibodies and dilutions: p-p44/42 MAPK (T202/Y204) (CST, #4370) (1:5,000), p44/42 MAPK (ERK1/2) (CST, #9102) (1:5,000), p-AKT (S473) (CST, #4060), AKT (pan) (CST, #4691) (1:5,000), p-CREB (Ser133) (CST, #9198) (1:5,000), CREB (CST, #9197) (1:5000), GAPDH (Novus Biologicals, #NB300-221) (1:10,000), IRDye 800CW Goat anti-Rabbit (Licor, #926-32211) (1:10,000), and IRDye 680LT goat anti-mouse (Licor, #926-68020) (1:10,000).

Table S1: List of significantly differentially translated genes from RiboTag microarray, see supplementary.xlsx file

Table S2: **List of antibodies**

**Flow cytometry antibody**	**Company and catalog number**	**Dilution**
CD62L (L-Selectin) Monoclonal Antibody (MEL-14), Brilliant Ultra Violet™ 737	Invitrogen, 367-0621-80	1/300
CD44 Monoclonal Antibody (IM7), Brilliant Ultra Violet™ 395	Invitrogen, 363-0441-80	1/400
BD Horizon™ BV711 Mouse Anti-GATA3 Clone L50-823	BD Biosciences, 565449	1/80
BD Horizon™ BV605 Rat Anti-Mouse CD4 Clone RM4-5 (also known as RM4.5)	BD Biosciences, 563151	1/133
BD Horizon™ BV421 Rat Anti-Mouse CD197 (CCR7) Clone 4B12	BD Biosciences, 566291	1/40
BD Pharmingen™ PerCP-Cy™5.5 Rat Anti-Mouse CD25 Clone PC61	BD Biosciences, 561112	1/100
CD3 Monoclonal Antibody (17A2), Alexa Fluor™ 488	Invitrogen, 53-0032-80	1/400
ROR gamma (t) Monoclonal Antibody (B2D), PE-Cyanine7	Invitrogen, 25-6981-80	1/800
Integrin alpha 4 beta 7 (LPAM-1) Monoclonal Antibody (DATK32 (DATK-32)), PE	Invitrogen, 12-5887-81	1/100
BD Pharmingen™ APC-Cy™7 Rat Anti-Mouse CD8a Clone 53-6.7	BD Biosciences, 561967	1/1000
IFN gamma Monoclonal Antibody (XMG1.2), APC	Invitrogen, 17-7311-81	1/800

**Primary antibody: Immunostaining/ immunoprecipitation**	**Company and catalog number**	**Dilution**
Rabbit anti Mouse Lyve1	Fitzgerald, 70R-LR005	1/500
Goat anti Mouse Lyve1	R&D Systems, AF2125	1/500
Goat anti Mouse VE-Cadherin	R&D Systems, AF1002	1/500
Rabbit anti Human/Mouse VE-Cadherin polyclonal	Abcam, ab33168	1/250
Rabbit anti Human/Mouse/Rat Madcam-1 Polyclonal	ThermoFisher, PA5-116979	1/200
Rabbit anti Human/Mouse/Rat Pentraxin3 Polyclonal	ThermoFisher, PA5-36156	1/200
Rabbit anti Human/Mouse CX47	ThermoFisher, 36-4700	1/200
Mouse anti Mouse/Rat/Guinea Pig/Human CGRP	Abcam, ab81887	1/200
Rat anti Mouse CD68	Bio-Rad, MCA1957	8 µg/mL

**Secondary antibody: Immunostaining**	**Company and catalog number**	**Dilution**
Donkey anti Rabbit AF488	ThermoFisher, SA5-10038	1/250
Donkey anti Rabbit AF594	JIR*, 711-585-152	1/500
Donkey anti Goat Cy3	JIR, 705-165-147	1/500
Donkey anti Goat AF647	JIR, 705-605-003	1/500
Donkey anti Mouse Cy3	JIR, 715-165-150	1/500

*Jackson ImmunoResearch

Supplemental Figure 1:

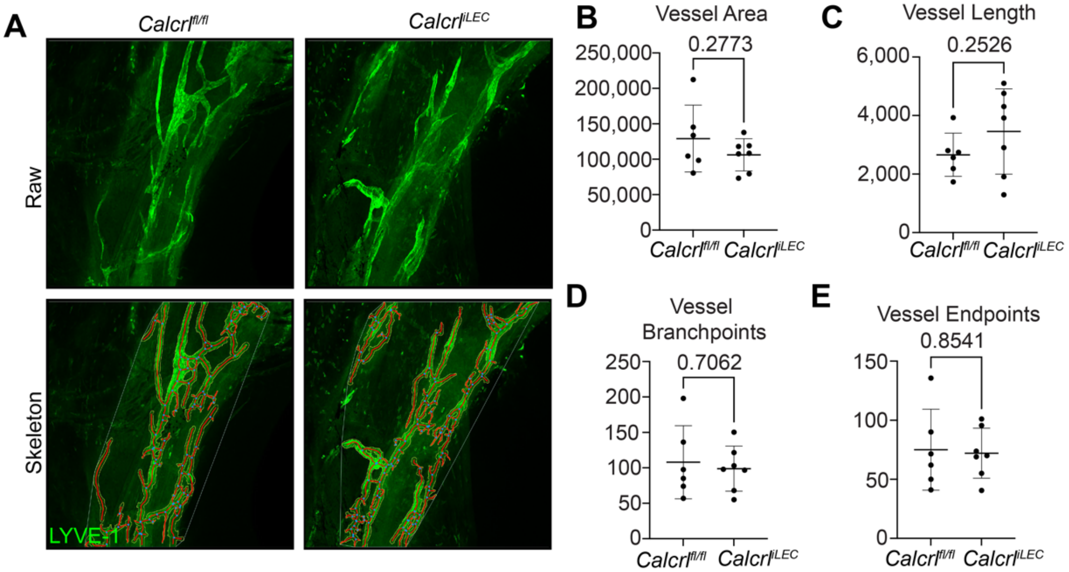

Supplemental Figure 1. Baseline characterization of *Calcrl^{iLEC}* meningeal lymphatic vessels 3 weeks following Tamoxifen induced cre-recombinase activation at the transverse sinus. (A) Representative images of *Calcrl$^{fl/fl}$* and *Calcrl^{iLEC}* meningeal lymphatic vessels (top) and AngioTool generated skeletonization of meningeal lymphatic vessels (bottom). Using AngioTool, total vessel area **(B)**, total vessel length **(C)**, vessel branchpoints **(D)**, and vessel endpoints **(E)** were assessed. N = 6-7 animals per group from 4 independent cohorts. Significance for C-E calculated using unpaired student's T Test. Graphs = mean ± standard deviation. All p values depicted.

Supplemental Figure 2:

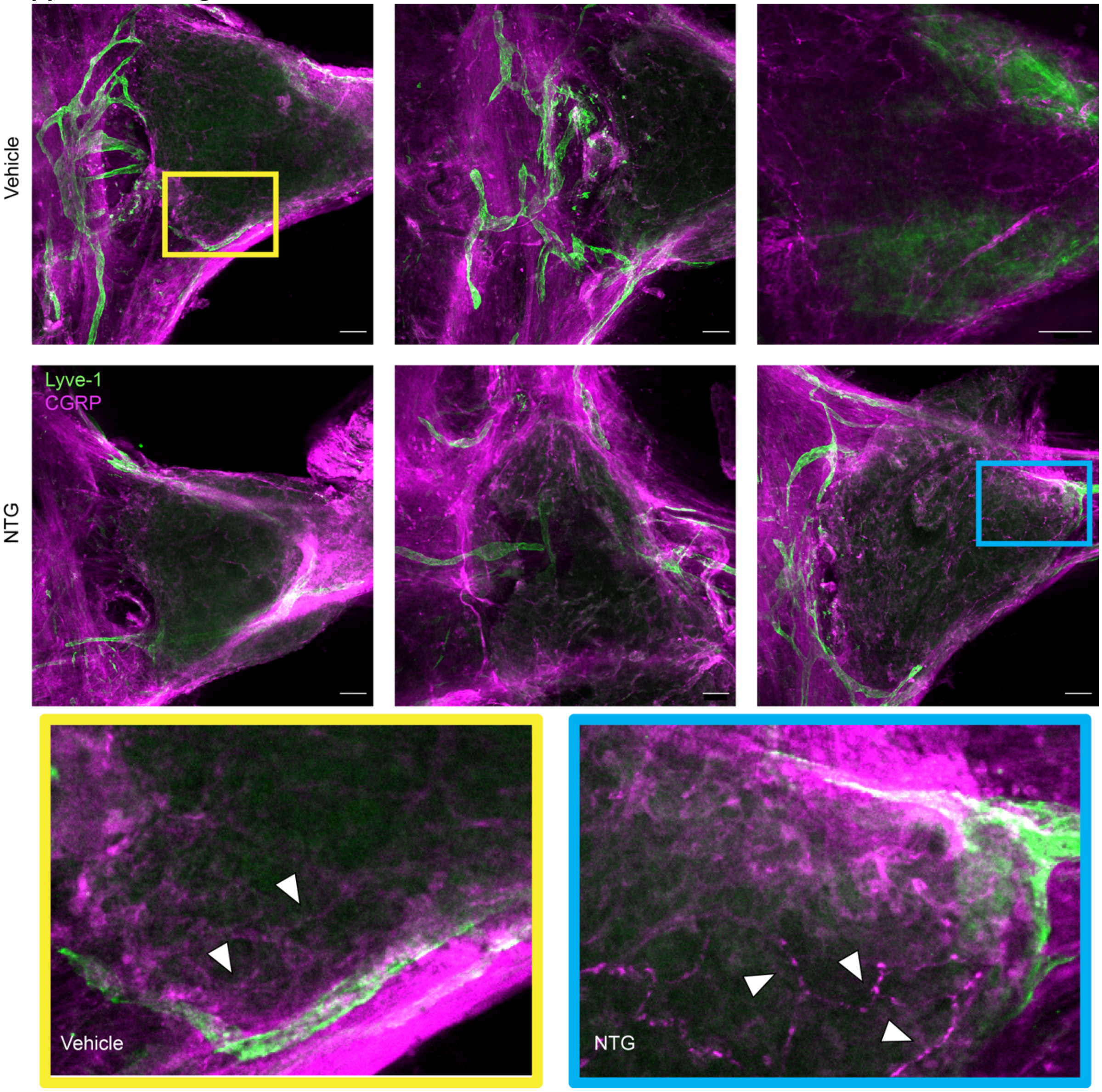

Supplemental Figure 2. Immunofluorescence microscopy of CGRP in mouse meningeal lymphatic vessels reveals close proximity of CGRP (magenta) positive neurons and Lyve1 (green) positive meningeal lymphatic vessels. Top Row: Vehicle treated mice. Middle Row: NTG treated mice. Bottom Row: Insets revealing increases in CGRP-positive nerves juxtaposed to meningeal lymphatics in NTG cohort. White arrows indicate likely CGRP positive axons in proximity to MLVs. N=3 mice per treatment group from one independent cohort. Scale bar = 100 um.

Supplemental Figure 3:

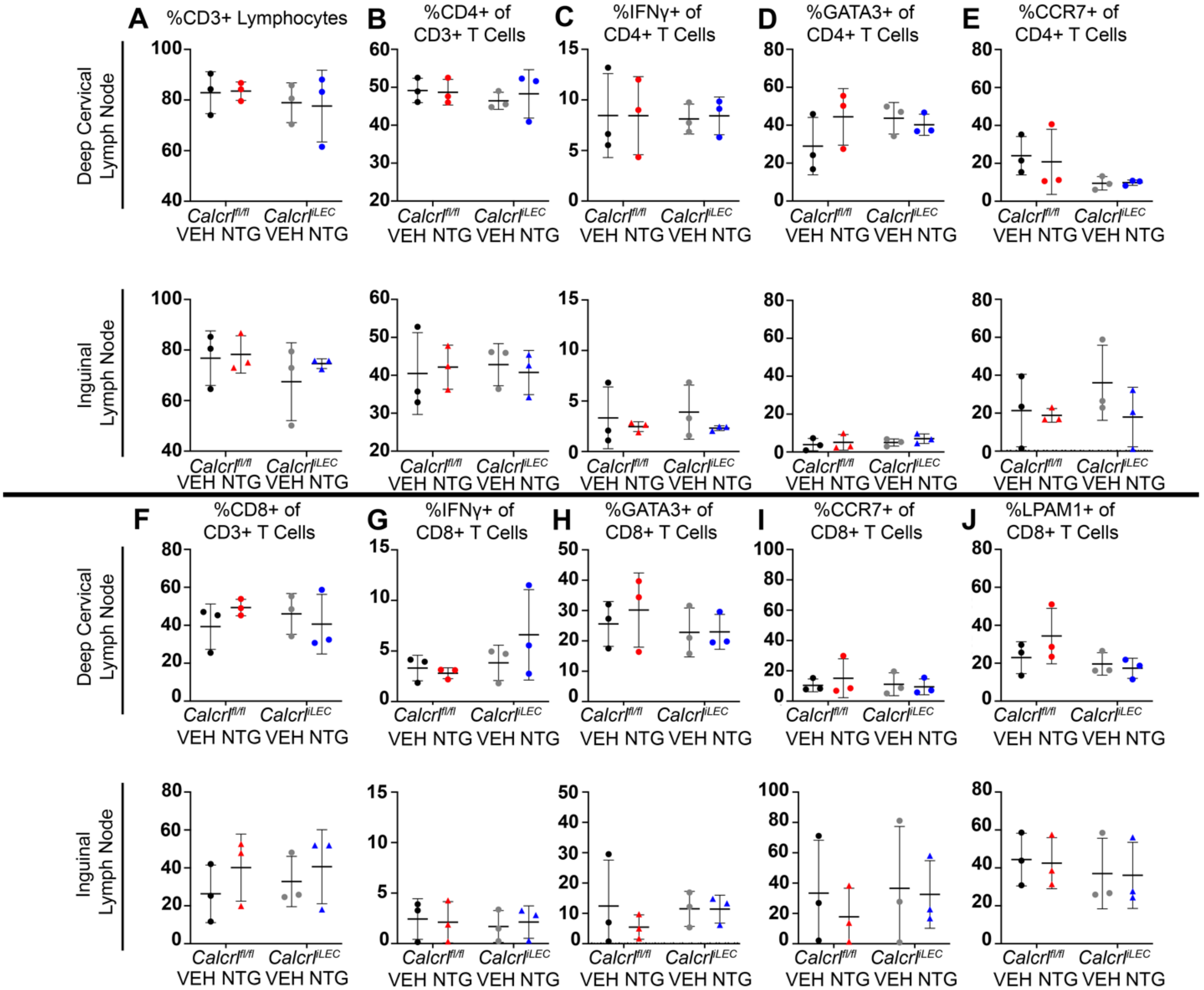

Supplemental Figure 3. Multispectral flow cytometry quantifying relative amounts of immune cells in deep cervical lymph nodes (Top) and inguinal lymph nodes (Bottom). (**A**) CD3+ T cells, (**B**) CD4+ T cells, (**C**) IFNγ+ CD4+ T cells (Th1 cells), (**D**) Gata3+ CD4+ T cells (Th2 cells), (**E**) CCR7+ CD4+ T cells (classical lymphatic homing), (**F**) CD8+ T cells, (**G**) IFNγ+ CD8+ T cells (Tc1 cells), (**H**) Gata3+ CD8+ T cells (Tc2 cells), (**I**) CCR7+ CD8+ T cells (classical lymphatic homing), **(J)** LPAM1+ CD8+ T cells (Madcam1 homing). N = 3 animals per group, pooled cells from left and right DCLN from two independent cohorts. Significance for each graph calculated using two-way ANOVA with Tukey's multiple comparison test. Graphs = mean ± standard deviation. P values depicted if < 0.05.
