# Supplementary material for: Meningeal lymphatic CGRP signaling governs pain via cerebrospinal fluid efflux and neuroinflammation in migraine models
Source: J Clin Invest. 2024 May 14;134(15):e175616. doi: 10.1172/JCI175616 (PMC11290972; doi:10.1172/JCI175616)
Supplement: Unedited blot and gel images [file jci-134-175616-s221.pdf]

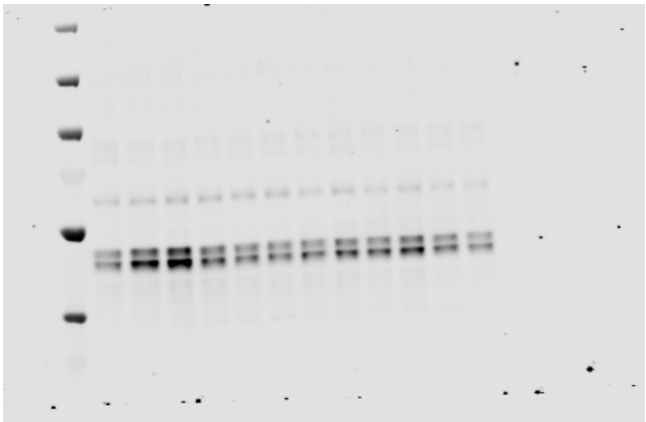

Full Unedited Gel for Figure 6

Phospho ERK; Replicate 1

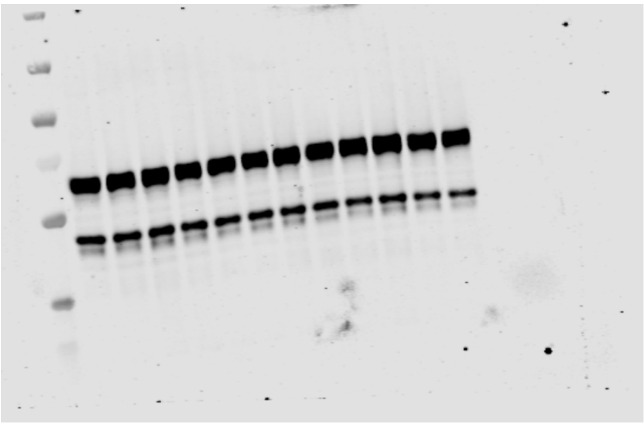

Full Unedited Gel for Figure 6

Total ERK; Replicate 1

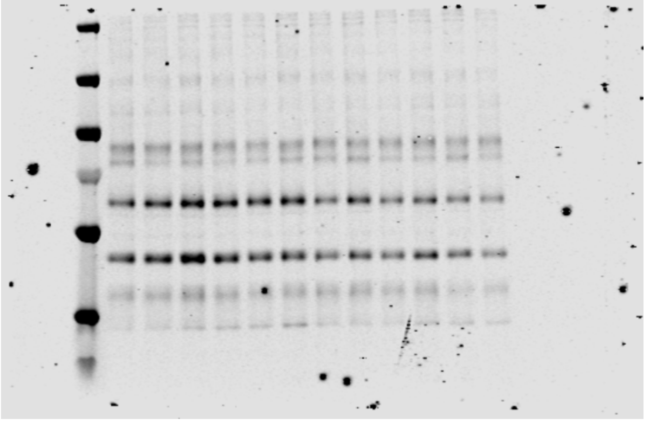

Full Unedited Gel for Figure 6

Phospho AKT; Replicate 1

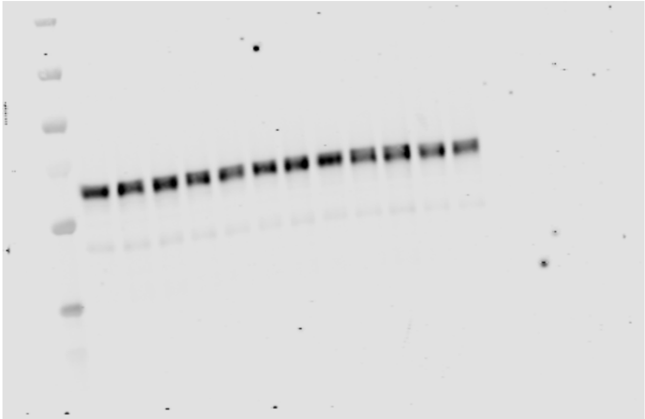

Full Unedited Gel for Figure 6

Total AKT; Replicate 1

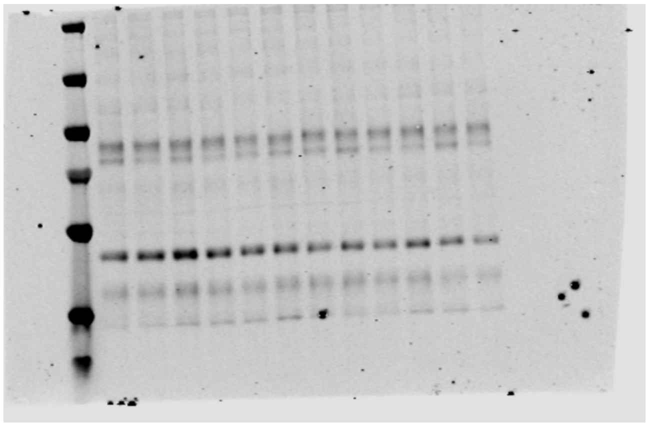

Full Unedited Gel for Figure 6

Phospho CREB; Replicate 1

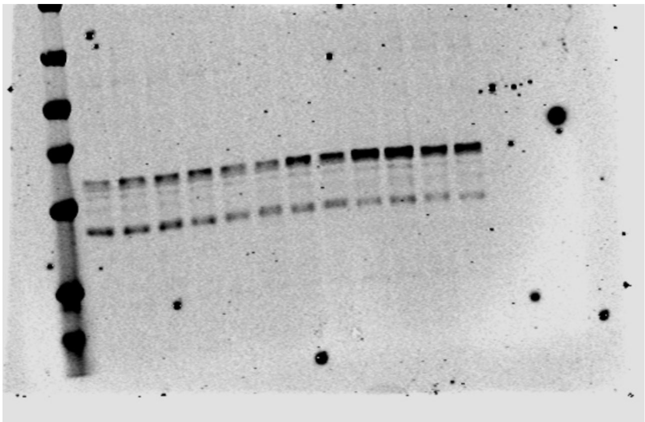

Full Unedited Gel for Figure 6

Total CREB; Replicate 1

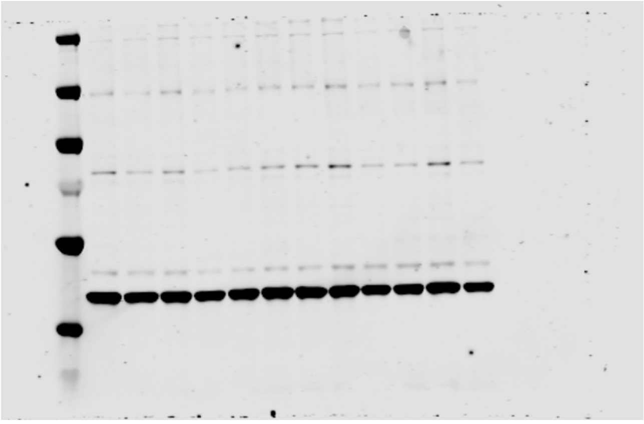

Full Unedited Gel for Figure 6

GAPDH_Phospho Blot; Replicate 1

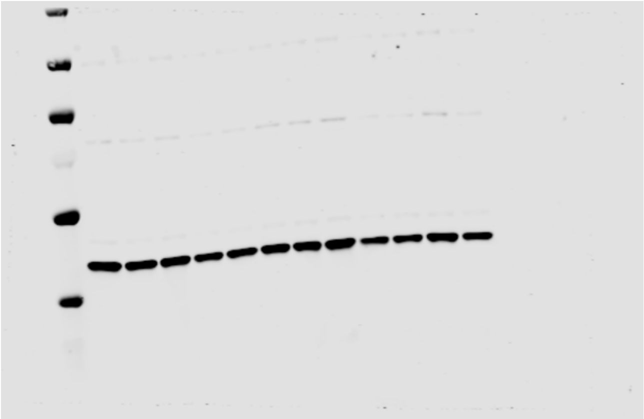

Full Unedited Gel for Figure 6

GAPDH_Total Blot; Replicate 1

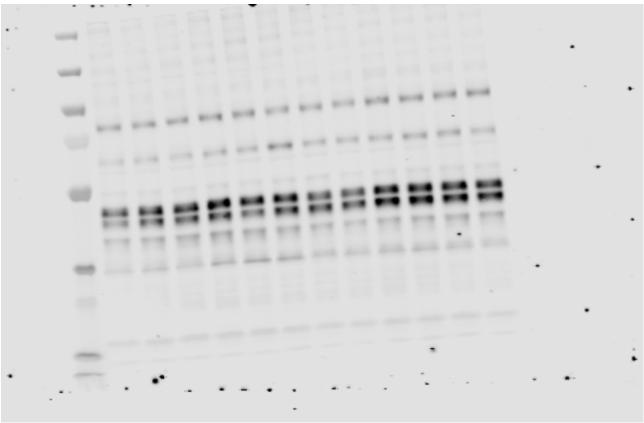

Full Unedited Gel for Figure 6

Phospho ERK; Replicate 2

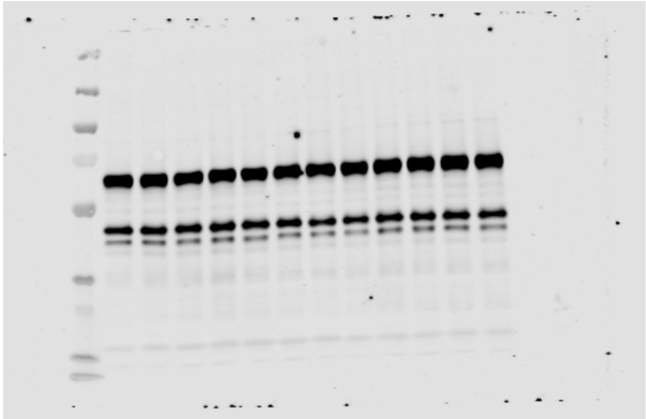

Full Unedited Gel for Figure 6

Total ERK; Replicate 2

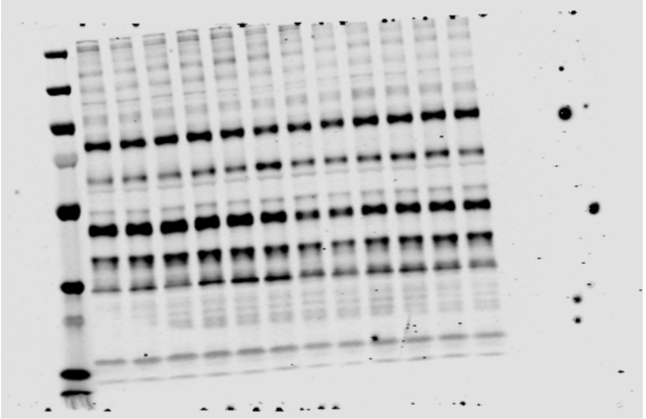

Full Unedited Gel for Figure 6

Phospho AKT; Replicate 2

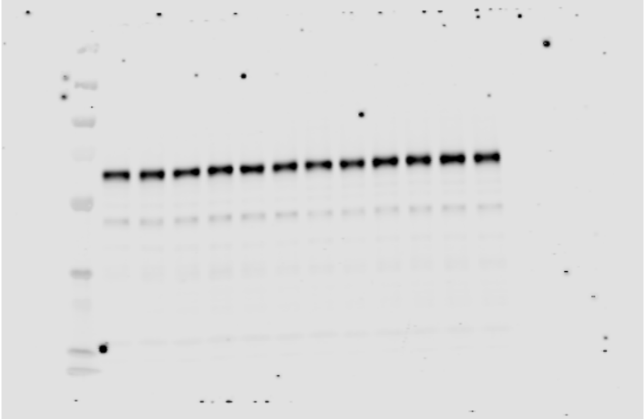

Full Unedited Gel for Figure 6

Total AKT; Replicate 2

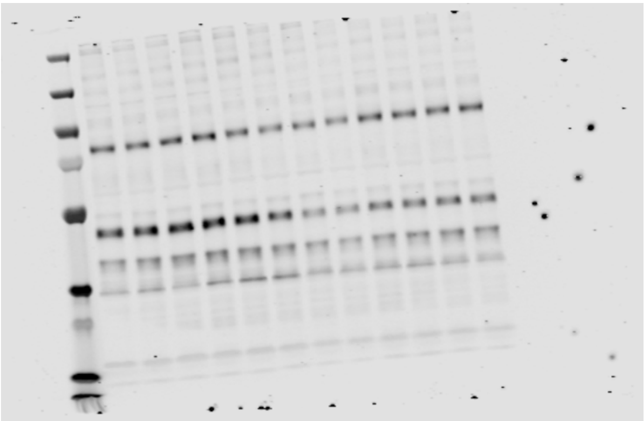

Full Unedited Gel for Figure 6

Phospho CREB; Replicate 2

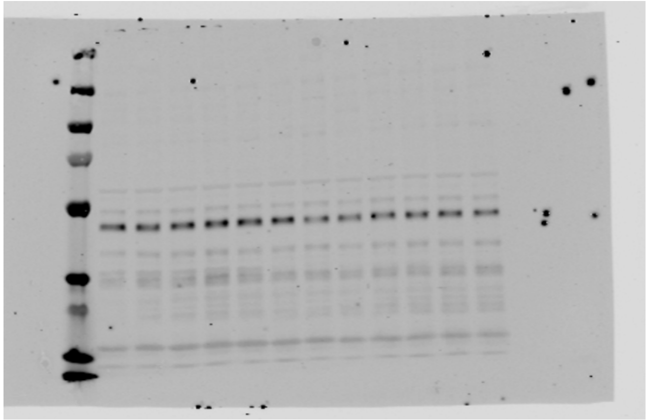

Full Unedited Gel for Figure 6

Total CREB; Replicate 2

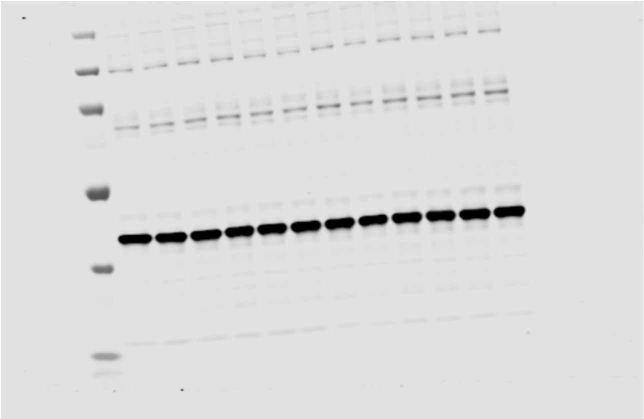

Full Unedited Gel for Figure 6

GAPDH_Phospho Blot; Replicate 2

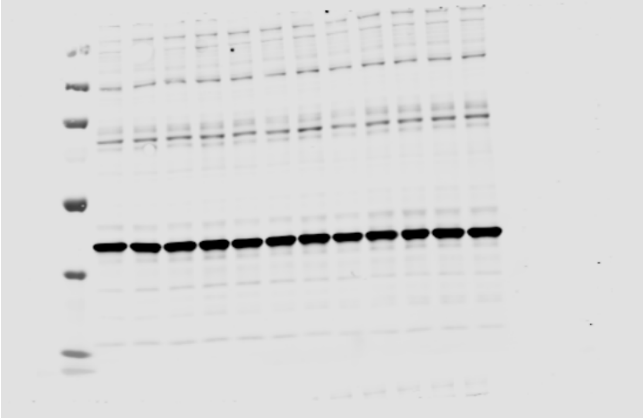

Full Unedited Gel for Figure 6

GAPDH_Total Blot; Replicate 2

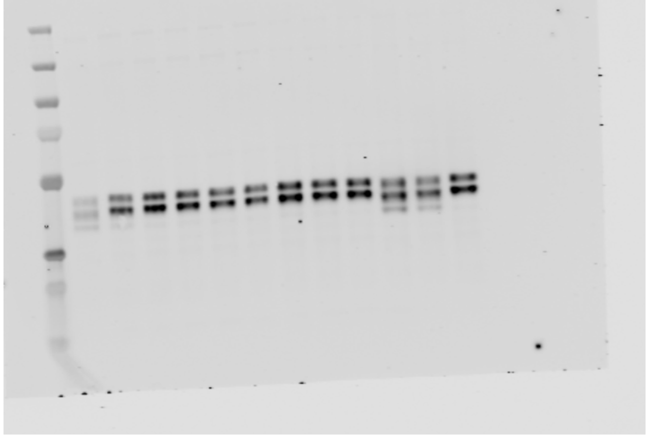

Full Unedited Gel for Figure 6

Phospho ERK; Replicate 3

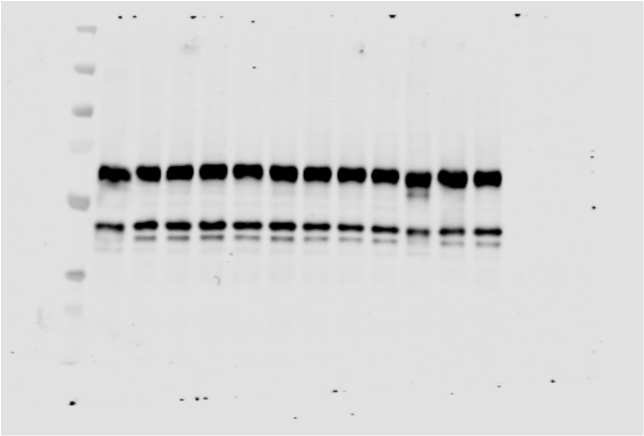

Full Unedited Gel for Figure 6

Total ERK; Replicate 3

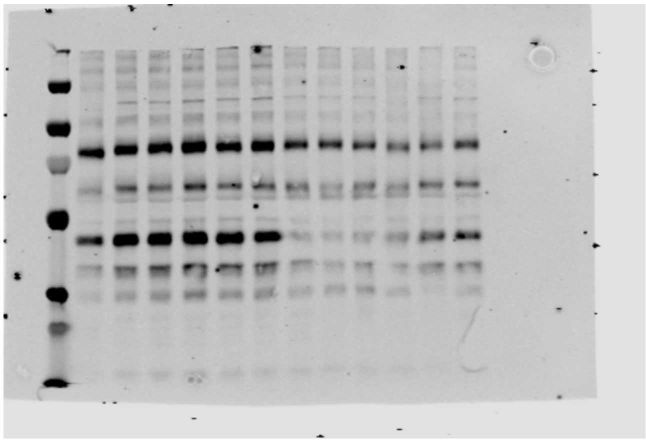

Full Unedited Gel for Figure 6

Phospho AKT; Replicate 3

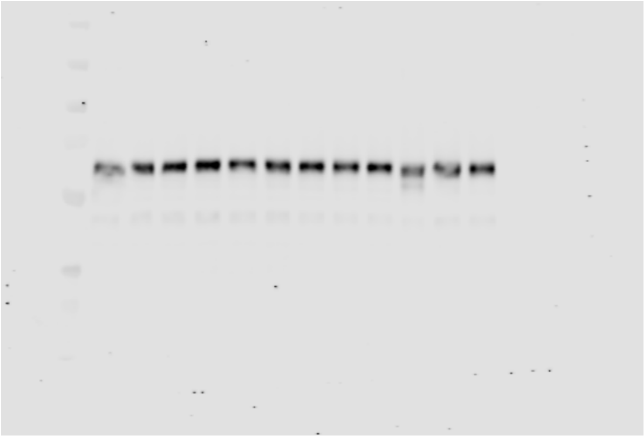

Full Unedited Gel for Figure 6

Total AKT; Replicate 3

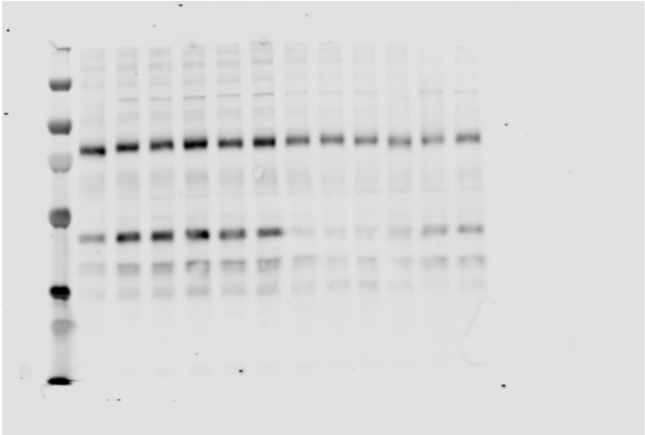

Full Unedited Gel for Figure 6

Phospho CREB; Replicate 3

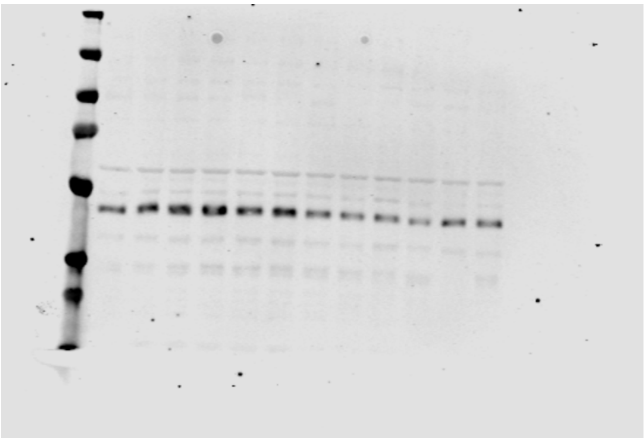

Full Unedited Gel for Figure 6

Total CREB; Replicate 3

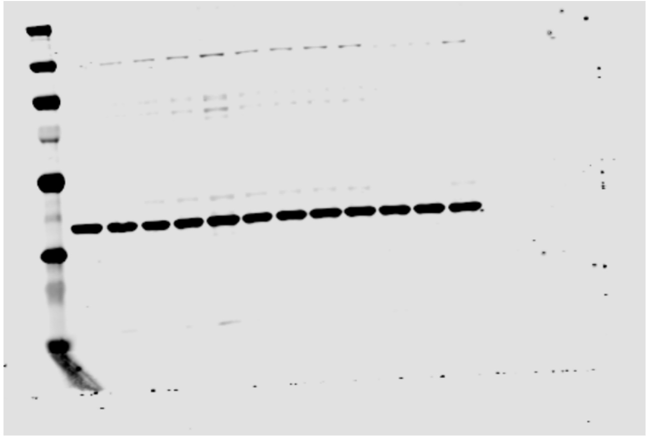

Full Unedited Gel for Figure 6

GAPDH_Phospho Blot; Replicate 3

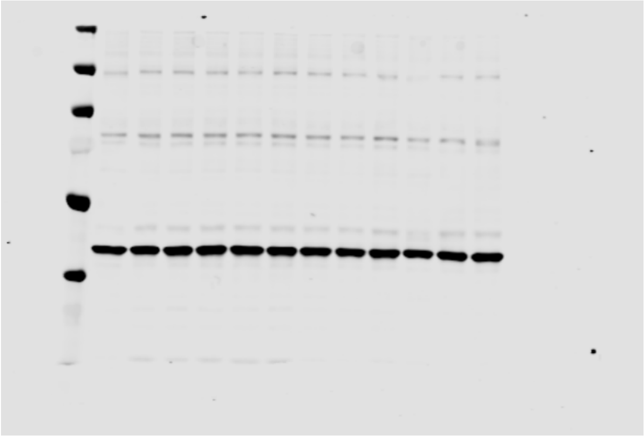

Full Unedited Gel for Figure 6

GAPDH_Total Blot; Replicate 3
